# NIRS-based neurofeedback training in a virtual reality classroom for children with attention-deficit/hyperactivity disorder: study protocol for a randomized controlled trial

**DOI:** 10.1186/s13063-016-1769-3

**Published:** 2017-01-24

**Authors:** Friederike Blume, Justin Hudak, Thomas Dresler, Ann-Christine Ehlis, Jan Kühnhausen, Tobias J. Renner, Caterina Gawrilow

**Affiliations:** 1 LEAD Graduate School & Research Network, University of Tübingen, Gartenstrasse 29, 72074 Tübingen, Germany; 2 Department of Psychiatry and Psychotherapy, University of Tübingen, Calwerstrasse 14, 72076 Tübingen, Germany; 3 Department of Psychology, University of Tübingen, Schleichstrasse 4, 72076 Tübingen, Germany; 4 Department of Child and Adolescence Psychiatry and Psychotherapy, University of Tübingen, Osianderstrasse 14-16, 72076 Tübingen, Germany; 5 Deutsches Institut für Internationale Pädagogische Forschung (DIPF), Schlossstrasse 29, 60486 Frankfurt/Main, Germany

**Keywords:** Attention-deficit/hyperactivity disorder, Neurofeedback, Biofeedback, Near-infrared spectroscopy, Electromyography, Virtual reality, Randomized controlled trial, School performance

## Abstract

**Background:**

Children with attention-deficit/hyperactivity disorder (ADHD) suffer from attention deficits, motor hyperactivity, and impulsive behaviour. These impairments are experienced at home, at school, and with friends. Functional imaging studies show that ADHD behaviour and impairments in executive functions (EFs) are mirrored by aberrant neurophysiological functioning. Moreover, several studies show that ADHD behaviour, impairments in EFs, and a lack of self-control contribute to poor school performance. Non-pharmacological interventions such as neurofeedback training (NFT), for instance, aim at improving neurophysiological and neuropsychological functioning as well as behaviour. Consequently, NFT is expected to improve school performance, EFs, and self-control in children with ADHD. Generalization of acquired self-regulation skills from laboratory to real life is crucial for a transfer to everyday situations and is hypothesized to be facilitated via training using virtual reality (VR) environments. Consequently, experiencing NFT in VR is expected to yield greater effects than training in two dimensions (2D).

**Methods/design:**

Ninety children with a clinical diagnosis of ADHD will be included in the study. Participants may be medicated or unmedicated. After random assignation to one of three conditions, all participants receive 15 training sessions of either near-infrared spectroscopy (NIRS)-based NFT in VR, NIRS-based NFT in 2D, or electromyogram-based biofeedback training in VR. ADHD symptoms, self-control, EF, health-related quality of life, school performance, and motor activity measured via parent, teacher, and child reports or objectively will be assessed before and after the intervention and at a 6 months follow-up. Furthermore, we are interested in parents’ expectations about the training’s effects.

**Discussion:**

This is, to our knowledge, the first study investigating the efficacy of NFT for children with ADHD in a VR compared to a 2D environment. Furthermore, this study will contribute to the discussion about the efficacy and specific and unspecific effects of NFTs in children with ADHD. In addition to commonly assessed variables such as ADHD symptoms, NIRS and behavioural data obtained in EF measures, health-related quality of life, and parents’ expectations about the intervention’s effects, this study will investigate the effects on self-control, school performance, and motor activity.

**Trial registration:**

ClinicalTrials.gov, NCT02572180. Registered on 19 November 2015.

**Electronic supplementary material:**

The online version of this article (doi:10.1186/s13063-016-1769-3) contains supplementary material, which is available to authorized users.

## Background

Children with attention-deficit/hyperactivity disorder (ADHD) are inattentive, hyperactive, and impulsive [1]. They also experience difficulties in waiting for rewards, planning actions, and self-controlling in situations characterized by delay [2–4]. Affecting 5% of all children worldwide, ADHD is one of the most prevalent mental disorders in children [5].

### School performance in children with ADHD

The core symptoms of ADHD, namely inattention, hyperactivity, and impulsivity, are present in various settings, for instance, when working on tasks that require sustained attention or while doing homework. Hence, ADHD affects performance levels at home and at school [1]. Children with ADHD demonstrate lower school achievement as a consequence of ADHD symptoms and concomitant impairments in executive functioning (EF) when compared to children without ADHD [6]. In addition, children with ADHD are four to five times more likely to be in need of special educational services compared to children without ADHD [7]. Several studies support the notion that the ADHD symptomatology acts as a primary reason for educational underachievement [7–9]. Several studies also provide evidence that deficits in EF such as, for instance, working memory and processing speed, might be crucially involved in impaired school performance of children with ADHD (see, e.g. [10–13]). Consequently, treatment of ADHD in schoolchildren should aim at improving behaviour as well as self-control and EF to eventually improve school performance.

### Neurophysiological findings in children with ADHD

Behavioural characteristics of children with ADHD are mirrored by altered cortical and subcortical activity patterns that can be measured with brain imaging techniques such as electroencephalography (EEG) and functional near-infrared spectroscopy (fNIRS) [14–16]. In EEG studies, children with ADHD show not only an increased theta/beta ratio, but also a reduced contingent negative variation (CNV) (see, e.g. [14, 17–19]). With fNIRS, Ehlis and colleagues [16] were able to provide evidence for a reduced concentration of oxygenated haemoglobin (oxy-Hb) in the ventrolateral prefrontal cortex of adults with ADHD, compared to a healthy control group, during a working memory task. This finding was replicated in children with ADHD for the inferior prefrontal cortex during a Stroop colour-word task [15]. These deviations from normal brain activity constitute neurophysiological correlates of behavioural problems and impaired EF in patients with ADHD (see, e.g. [14, 16, 20, 21]). Consequently, we assume a treatment aiming at normalizing these deviant neurophysiological patterns to improve behaviour and EF in children with ADHD.

### Neurofeedback training (NFT) in children with ADHD

Neurofeedback training (NFT) sessions are interventions based on the above-mentioned neurophysiological findings. They aim at improving self-regulation on two levels: on a neurophysiological as well as on a cognitive behavioural level [22]. In NFT, brain activity is translated into simple visual or acoustic signals which are immediately fed back to the patient [23]. Depicting learning as a controlled, effortful, and explicit as well as implicit, automatic process that is influenced by cognitive-attributional variables such as motivation, allows patients to acquire techniques that allow them to self-regulate their brain activity [22]. Hence, NFT aims at facilitating phasic changes of brain activity and enhancing neurophysiological functioning [22]. In addition, NFT aims to improve self-regulation on cognitive behavioural levels; i.e. participants are required to concentrate, to sit still, to endure boredom, and not to react on impulse during the training sessions.

Studies examining the effects of EEG-based NFT show inconsistent results. For instance, Holtmann and Cortese and colleagues [24, 25] could not find evidence for an improvement of ADHD symptoms that was specifically related to the NFT itself. However, Arns and colleagues [26] found significant effects when comparing 15 studies in a meta-analysis. Furthermore, Marx and colleagues [27] showed in a pilot study that NIRS-based NFT in children with ADHD significantly reduced ADHD symptomatology after 12 training sessions. Extending beyond a mere influence of NFT on ADHD symptomatology, Meisel and colleagues [28] demonstrated that NFT significantly improved academic performance in children with ADHD. In contrast, stimulant medication could not be shown to effectively help schoolchildren in overcoming poor school performance, although it has a significant effect on improving behaviour [29]. However, further research is required to clarify the effects of NFT, especially NIRS-based NFT. Besides effects on ADHD symptoms, school performance, EF, EF-related frontal lobe activation, health-related quality of life (HRQoL), and self-control, the present study will investigate potential moderating influences of baseline ADHD symptoms, self-control, and IQ as well as training motivation. The effects of two NIRS-based NFT types (see below) will be compared to effects from an active control condition receiving an electromyogram (EMG)-based biofeedback training (BFT).

### Control conditions for NFT studies

In prior NFT research, different control conditions have been used to investigate the efficacy of NFTs. For instance, sham feedback has been implemented, but is criticized due to strong ethical concerns and participants’ poor compliance to treatment [30–33]. In the present study, an active control condition receiving an EMG-based BFT will be used to illustrate specific as well as unspecific effects of NIRS-based NFT. Looking at the effects of NFT and BFT, it is important to recall that NFT aims at improving self-regulation on two levels, neurophysiological as well as cognitive behavioural [22]. As illustrated below, the latter level is also targeted in BFT. In both NIRS-based NFT and EMG-based BFT, participants are expected to acquire self-regulation skills that allow the exertion of control over a specific endogenous parameter, for instance, prefrontal activity in the NFT and activity in the musculi supraspinatus in the EMG condition. In addition, participants learn to self-regulate behaviour such as being attentive, sitting still, not reacting on impulse, and enduring boredom. Consequently, we expect both NIRS-based NFT and EMG-based BFT to yield similar behavioural effects, as participants learn to self-regulate behaviour in both conditions. However, effects related to the acquisition of self-regulation skills related to the respective endogenous parameter are uniquely attributable to the parameter itself. As only NIRS-based NFT aims at normalizing aberrant brain activity, which is assumed to constitute a neurophysiological correlate of behavioural problems in children with ADHD [15], we consequently expect larger total effects from the NIRS-based NFT than from the EMG-based BFT.

### NFT in a virtual reality (VR) environment

To our knowledge, until now, no NFT study in children or adults with ADHD employing a virtual reality (VR) environment as a training setting has been conducted. However, from our perspective, there are several reasons suggesting that patients with ADHD can profit from training in a VR environment.

First, it is hypothesized that both the acquisition of self-regulation skills in the laboratory and the transfer to everyday life situations (e.g. a classroom setting) will be facilitated by training in a naturalistic VR environment. VR environments are often used in the treatment of mental disorders such as anxiety disorders and post-traumatic stress disorder, and were shown to be equally effective compared to therapies employing exposures to real-life situations [34]. Strong effects of therapies using naturalistic VR environments are attributed to the fact that various naturalistic stimuli, i.e. sounds, visual impressions, and haptic experiences, stimulate different sensory channels at once, thereby eliciting realistic psychological and behavioural responses [34]. Consequently, children with ADHD are expected to behave similarly inattentively, hyperactively, and impulsively in VR as well as in real-life classrooms. In NFT and BFT sessions, therapists may use these responses to work towards changes in behaviour by correcting inadequate, and by reinforcing appropriate behaviour, i.e. by training successful self-regulation of behaviour. Furthermore, aberrant psychological responses occurring in specific situations, for instance, an underactivation of prefrontal cortical areas in children with ADHD, are elicited by a naturalistic VR environment such as a VR classroom [34]. In NFT, but not in an EMG-based BFT, these inadequate responses are corrected as participants acquire self-regulation strategies that allow them to normalize their brain activity. Additionally, the effects of therapies employing naturalistic VR environments can be attributed to the high degree of realism that supports the transfer of skills acquired in the therapy or training to real-life situations, i.e. from a VR to a real-life classroom [34]. Consequently, we expect larger effects from NFT taking place in naturalistic VR environments than from training taking place in two-dimensional (2D) settings, as the acquisition and transfer of behavioural and psychological self-regulation skills are facilitated.

Second, after reviewing predictors and moderators of the efficacy of cognitive training, Keshavan and colleagues suggest that training motivation plays a major role [35]. This is in line with results presented by Käthner and colleagues, who provide evidence for a significant influence of motivation on task performance in the brain-computer interface [36]. The crucial role of training motivation in making cognitive training effective can be explained by findings that support the assumption that motivational state and positive mood facilitate prefrontal activation and consequently cognitive control, that is “the ability to select thoughts or actions in relation to internal goals” [37, 38]. In NFT for children with ADHD, both cognitive control and variability in prefrontal activity are essential, as participants are instructed to select thoughts that allow for a self-regulated increase or decrease of prefrontal activity. Consequently, NFT should aim at creating training settings that foster training motivation and positive mood. According to Keshavan and colleagues, intrinsic motivation in cognitive training can best be fostered by providing a “personalized context that links cognitive training to goals of everyday life” [35]. With the naturalistic VR classroom of the present study, a personalized context of everyday life is provided and should consequently foster cognitive control and prefrontal activation of the participants. Consequently, we expect NFT and BFT taking place in a naturalistic VR environment to yield larger effects than training in 2D. Furthermore, the effects of the training are expected to be moderated by the training motivation.

### Hypotheses

First, we hypothesize that NIRS-based NFT of the frontal lobe (dorsolateral prefrontal cortex (dlPFC) and EMG-based BFT improve ADHD symptoms, self-control, EF, HRQoL, school performance, and motor activity in children with ADHD independent of whether the training is conducted in 2D or VR. Second, we expect larger positive effects for NIRS-based NFT in 2D and VR than for EMG-based BFT in VR both at a post-test time point and at 6 months follow-up. Third, we expect the effects of NIRS-based NFT to be larger in the VR condition. Fourth, for NIRS-based NFT in 2D and VR, we expect an increase prefrontal in cortical activation during EF tasks at post-test and at 6 months follow-up.

## Methods/design

This manuscript as well as the trial it describes are in accordance with the Standard Protocol Items: Recommendations for Interventional Trials (SPIRIT) guidelines [39, 40]. See Additional file 1 for the SPIRIT checklist.

### Participants and recruitment

We will recruit approximately 90 participants with a clinical diagnosis of ADHD (any presentation) that is given based on the 5th edition of the *Diagnostic and Statistical Manual for Mental Disorders* [41] via medical offices of paediatricians, child and youth psychologists, and psychiatrists as well as offices of occupational therapists. Furthermore, we will recruit participants via the outpatient department of the Department of Child and Adolescent Psychiatry and Psychotherapy, University Hospital Tübingen, and local school psychologists. In addition, circular emails sent to members of the University of Tübingen, websites of the authors’ departments, local newspapers, and radio stations will announce the study. Information gained using the long version of the Conners 3 questionnaire for teachers and parents [42], the Strengths and Difficulties Questionnaire **(**SDQ-Deu) [43], and an interview with the parents are used to confirm diagnoses of ADHD. The training sessions will take place in the Department of Psychiatry and Psychotherapy at the University of Tübingen. See Table 1 for an overview of the eligibility criteria.

**Table 1:** Eligibility criteria

Inclusion criteria	In school Grades 1–4 (age 6–10).
	Clinical diagnosis of ADHD combined, predominantly inattentive or predominantly hyperactive-impulsive presentation according to the Diagnostic and Statistical Manual of Mental Disorders, fifth edition (DSM-5)
	Written informed consent from parents/legal guardian
Exclusion criteria	IQ <70 as assessed with the Culture Fair Test (CFT) 1-R or the CFT 20-R [44, 45]
	Parent-reported diagnosis of the following: serious physical illness or chronic diseases such as pulmonary diseases, heart diseases, diabetes, hypertension, and rheumatic diseases; neurological disorders including stroke, multiple sclerosis, and epilepsy; indicated psychiatric disorders including obsessive-compulsive disorder, chronic tic disorders, Tourette’s syndrome, and suicidal behaviour
	Prior or current participation in neurofeedback training (NFT)/biofeedback training (BFT)
	Other psychotherapeutic treatment or any kind of attention training, also in the course of an ergotherapeutic treatment, while participating in the study

### Randomization

The design involves three conditions (*n* = 30 per condition; *N* = 90) to which recruited children will be assigned randomly upon confirmation of all inclusion criteria. One of the principal investigators of this study who is only occasionally involved in training and testing participants executes the randomization. A block randomization procedure is applied, and balancing the conditions for age, gender, and ADHD medication stratifies the randomization.

### Interventions

Two conditions involve 15 sessions of a NIRS-based NFT, one in a VR classroom setting and one in a 2D classroom setting to control for specific effects of trainings in VR and 2D. The third condition involves an EMG-based BFT in VR and constitutes a control condition that allows the evaluation of effects that are uniquely attributable to the NFT itself.

Every training session lasts approximately 60–70 min including a preparation phase at the beginning (20 min), in which the NIRS cap and optodes are fitted to the head, or the EMG electrodes are placed on both musculi supraspinatus and both mastoids. For the participants in the VR classroom setting, the head-mounted display (HMD) is mounted. The training sessions also include the NFT or BFT (45 min) and a training phase with stimulus cards at the end of the training sessions 6–15 (5 min). The stimulus cards present a screenshot of the 2D screen. Within the laboratory setting, these cards are introduced as cue stimuli associated with brain activation or increased muscular activity of the musculi supraspinatus as learned during the NFT or BFT. When employed at home, they are thought to facilitate activation and to establish an association between, for instance, doing homework and brain or muscle activation [44].

Every training session with NIRS-based NFT or EMG-based BFT consists of three blocks, of which the first and the second are with continuous performance feedback (feedback condition). In the third block, no contingent performance feedback is provided (transfer condition), which is thought to foster generalization of acquired self-regulation skills to real-life situations [45]. For the NIRS-based NFT, the first and the second blocks consist of 12 trials, the third of 8. Each trial starts with an active phase of 30 s in which the respective endogenous parameter, that is oxy-Hb in the bilateral dlPFC for NIRS-based NFT, should be regulated and is followed by a resting phase of 20 s at the end. For the EMG-based BFT, the first and the second block consist of 24 trials; the third block consists of 16 trials. Each trial starts with an active phase of 15 s in which muscular activity of the musculi supraspinatus should be regulated and is followed by a resting phase of 10 s. Durations of active and resting phases in NIRS-based NFT and EMG-based BFT conditions vary due to different response times of the respective endogenous parameters towards the beginning of self-regulation processes employed by the participants [46, 47]. Among the conditions, the amounts of trials are varied in order to obtain an equal total training duration for all conditions.

Lighting in the VR and 2D classroom provides the feedback. For the NIRS-based NFT, lighting increases with increasing activity in the bilateral dlPFC, i.e. increasing oxy-Hb, and decreases with decreasing activity, i.e. decreasing oxy-Hb. Lighting for the EMG-based BFT increases with increasing muscular activity in the right musculus supraspinatus compared to the left and decreases with increasing activity in the left musculus supraspinatus compared to the right.

During the first eight training sessions, the training follows a protocol with 50% activation and 50% deactivation trials. For the second half of the training sessions, the protocol changes to 80% activation and 20% deactivation trials. At the beginning of each trial, an arrow appearing on the blackboard of the VR or 2D classroom pointing upwards indicates an activation trial, while an arrow pointing downwards indicates a deactivation trial.

**Fig. 1:**
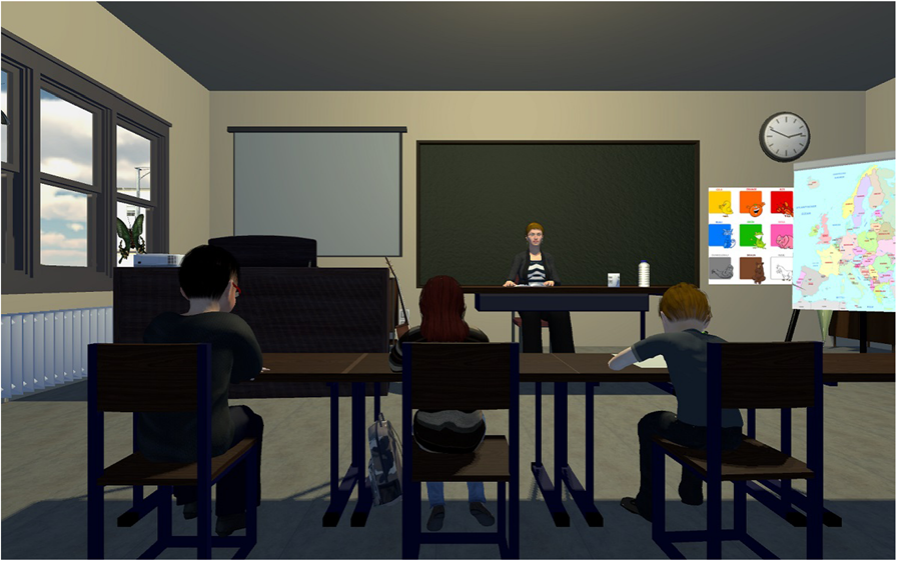
2D and VR classroom

**Fig. 2:**
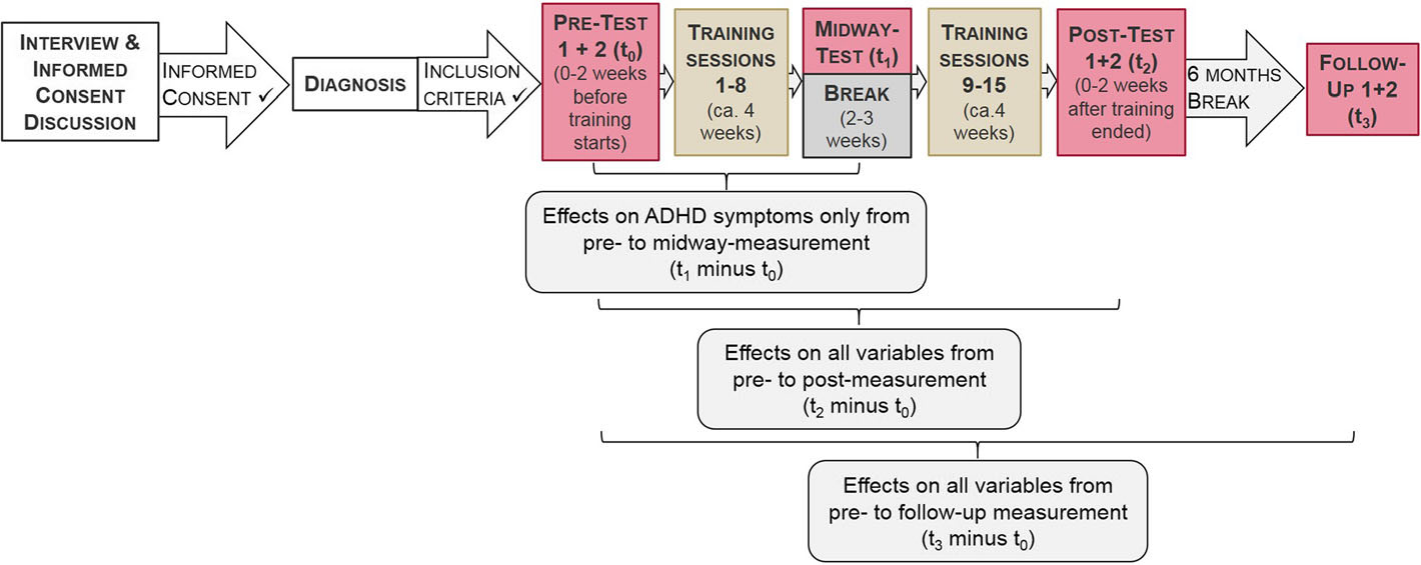
Flow chart showing the course of the study

After eight training sessions, participants have a break of 2 to 3 weeks that should further support transfer to real-life settings by using stimulus cards with screenshots of the training setting. The stimulus cards show the image of the classroom that participants see during the training with the arrow pointing upwards and will be introduced in the laboratory setting at the end of training session numbers 6, 7, and 8. Participants are instructed to look at the cards, employ activation strategies they learned during the training, and imagine increasing the lighting five to six times. After this activation task, they solve a riddle appropriate for their age and knowledge. For the break, participants are instructed to practice activation at home at least once per day prior to a situation that requires sustained attention, e.g. doing homework. For the rest of the training sessions, participants are asked to continue practicing activation at home. Furthermore, they still practice with the cards at the end of every training session. The 2D and VR classroom is shown in Fig. 1. An overview of the study course is presented in Fig. 2.

### Positive reinforcement

In both NFT and BFT, an animated teacher in the VR or 2D classroom provides reinforcement via positive auditory feedback if the participant performed successfully in the past trial. In addition, smileys appear on the blackboard of the classroom to provide positive reinforcement at the end of every successful trial. Success is calculated as follows. For the NIRS-based NFT, a baseline is calculated as the average oxy-Hb signal from the eight dlPFC channels (four on the left and four on the right hemisphere) in the last 5 s before the start of each trial. For the EMG-based BFT, the baseline is calculated as the average normalized EMG output (right musculus supraspinatus EMG output minus left musculus supraspinatus EMG output) in the last second before the start of each trial. Reinforcement is provided with one smiley when the participant has spent 60–69% of the time of the second half of the trial on the required side of the baseline. For the NIRS-based NFT, below the baseline is a decrease in the oxy-Hb signal from the eight dlPFC channels, and above is an increase in the oxy-Hb signal from the eight dlPFC channels. For the EMG-based BFT, below the baseline is an increase in the activity in the left musculus supraspinatus compared to the right, and above is an increase in the activity in the right musculus supraspinatus compared to the left. Analogously, the participant receives two smileys with 70–79% and three smileys with at least 80% of the second half of the trial spent in the required direction. Furthermore, reinforcement for the second block changes adaptively with the performance in the first block. If the participant scored between 40 and 60% success rate in the first block, the second block will remain exactly like the first. If the participant achieves lower than a 40% success rate, the threshold will decrease to .8 standard deviations (*SD*) in either direction relative to the baseline, so that fluctuations in the light are more sensitive to performance. In addition, the threshold for receiving smileys would decrease to 50%, 60%, and 70% of the time that must be spent on the required side of the baseline, for one, two, or three smileys, respectively. If the participant scores higher than a 60% success rate in the first block, the threshold increases to 1.2 *SD* above and below the baseline, hence making changes in the lighting, requiring more relative activation or deactivation. In addition, the baseline is artificially augmented to be .1 *SD* above or below the calculated baseline. Consequently, in order to receive smileys, the participant has to maintain a stronger activation or deactivation than before. The third block will be calculated in the same way as the second.

### The VR and 2D classroom

In the VR and 2D classroom, every participant is seated at a virtual table in the second row of a primary school classroom (see Fig. 1.). Other pupils surround him/her, and a teacher sits in the front of the classroom at a desk. Visual, auditory, and mixed distractors such as, for instance, paper planes flying through the room, fellow students whispering, or people knocking on the door will be randomized to occur in 50% of all trials. Their appearance is balanced between trials and sessions, and the distractors appear with a distance of at least 60 s between two distractors.

### Token system

Children are rewarded for their participation. At the beginning of the study, they receive a sticker album and one sticker per test or training session in the course of the study. If participants report to have trained regularly with the stimulus cards during the break, they can earn two more stickers.

### Adverse events

Reported adverse events and other unintended effects of the interventions employed in this study or the trial conduct are recorded and discussed with psychologists as well as child and youth psychiatrists.

### Assessments

#### Culture Fair Intelligence Test 1-R and 20-R

The Culture Fair Intelligence Test 1-R (CFT 1-R) [48] is a non-verbal intelligence test that can be used for children aged 5 years and 3 months to 9 years and 11 months. It consists of five subtests on substitutions, mazes, classifications, similarities, and matrices. The test can be applied in a short and a long form that differ in testing time only, but not in the amount of subtests applied. The short form will be used in this study. Reliability scores for the subtests vary between *r* = .75 and *r* = .90, and reliability for the short form is reported to be *r* = .94. Retest reliability with a second measurement after 2.5 months is *r* = .90 [48]. For children aged 8 years and 5 months and older, the Culture Fair Intelligence Test 20-R (CFT 20-R) [49] is used. The CFT 20-R is a non-verbal intelligence test consisting of two parts, each containing four identically constructed subtests on completing series, classifications, matrices, and topological reasoning. In this study, the test is applied in its short version, which consists of only the first of the two parts. The reliability of the CFT 20-R is *r* = .92 for the short version of the test. The retest reliability is adequate, with *r* = .85 after 2 months [49]. Hence, both tests show adequate psychometric properties to measure intelligence in the study sample.

#### Conners 3rd Edition (Conners 3) - German translation

The German long versions of the Conners 3 for parents (C3-P) and teachers (C3-T) consist of 108 items for parents and 112 items for teachers. The Conners 3 tools assess ADHD symptoms but also learning problems, EF, peer relationships, and aggression/defiance [42]. Furthermore, the questionnaires contain screener items for anxiety and depression. Both versions of the Conners 3 have adequate psychometric properties for teachers and parents with good internal consistency for most of the scales (Cronbach’s α > .85) and acceptable values for the remaining scales (Cronbach’s α > .70). Test-retest reliabilities are also good, with average values of about *r* = .85. Consequently, the Conners 3 in its German version seems to be appropriate to assess the effects of the intervention administered in this study.

#### Strengths and Difficulties Questionnaire

The Strengths and Difficulties Questionnaire (SDQ) consists of 25 items and assesses behavioural strengths and difficulties of children on five scales: prosocial behaviour, hyperactivity, emotional problems, peer relationships, and conduct problems [43]. Different versions for teachers and parents are available and are used in this study. The factorial structure of the original English questionnaires was also found for the German translations (SDQ-Deu) [50]. Psychometric properties for the German versions are good, with high internal consistency for the whole questionnaire (Cronbach’s α = .82) and at least acceptable scores for the subscales (Cronbach’s α between .58 and .76). The retest reliability is specified with *r* = .62 [51]. Consequently, the SDQ-Deu is an appropriate measure to assess effects of the therapeutic intervention of this study.

#### KINDL-R

The KINDL-R questionnaires assess health-related quality of life (HRQoL) via self-report and parent rating on six scales: physical and mental well-being, self-esteem, family, friends, and functional capability in daily life at school. In this study, the Kid-KINDL-R for children aged 7–13, as well as the Kiddo-Kindl-R for parents of children aged 7–13, are applied. Psychometric quality and overall consistency of the parent questionnaire are good, with Cronbach’s α = .85 for the total scales and values ranging between α = .63 and α = .71 for the subscales [52]. Likewise, psychometric quality of the self-report questionnaire for children is good, with Cronbach’s α = .82 for the total scales and values between α = .54 and α = .73 for the subscales. Hence, the KINDL-R questionnaires constitute a suitable instrument to measure HRQoL in this study.

#### Brief Self-Control Scale (SCS-K-D)

The German brief version of the Self-Control Scale (SCS-K-D) assesses self-control using 13 items [53]. The SCS-K-D in the version presented by Rauch and colleagues [53] assesses self-control via parent report. With a retest reliability of *r* = .82, the psychometric quality is good. Adding to the parent report, we adapted the questionnaire to a self-report measure that can be used with children. Piloting the adapted version of the self-report questionnaire for children, we confirmed its psychometric quality, as internal consistency was high, with Cronbach’s α = .80. Consequently, the SCS-K-D is a suitable instrument to assess self-control capacity in the study sample.

#### Questionnaire on academic self-efficacy

Academic self-efficacy is a concept describing expectations about competences that will be exhibited when confronted with academic demands. These expectations are often described from the students’ own perspectives. The self-report used in this study questionnaire consists of seven items, and the internal consistency varies between Cronbach’s α = .70 and .73 due to different measurements [54]. We reworded the items and piloted them in 34 children aged 8–10. Internal consistency of the adapted scale was similar to the original scale with Cronbach’s α = .71. Although psychometric quality is only acceptable, this measure is regularly and successfully used to assess self-efficacy in children (see, e.g. [55]).

#### Behaviour Rating Inventory of Executive Function (BRIEF)

The Behaviour Rating Inventory of Executive Function (BRIEF) is a set of questionnaires that assess executive functions of children aged 6–16 (parent and teacherreports) and 11–16 (self-report) [56]. For this study, only parent and teacher reports are applied. These questionnaires contain 86 items that load on eight subscales of two main indices. The index ‘behaviour regulation’ subsumes the subscales *inhibition, shifting,* and *emotional control*. The index ‘cognitive regulation’ comprises the subscales *initiate, working memory, plan/organize, organization of materials,* and *task-monitoring*. The internal consistency of the teacher and parent questionnaires is very good, with values between α = .79 and α = .98 [56]. The retest reliability for the parent questionnaires is adequate, with values higher than *r* = .80 for most of the scales. The values are more than *r* = .90 for the teacher questionnaires.

#### Parents’ expectations about the training’s effects

Parents’ expectations about the training’s effects are assessed using the scale *expectations of changes* of the Fragebogen zur Erfassung relevanter Therapiebedigungen (FERT), a questionnaire that assesses relevant therapy conditions [57], in an adapted format. The scale consists of eight items and has been adapted from reporting about a person’s own experiences of his or her therapy to reporting about an intervention that is experienced by the child of the reporting person. The factorial reliability of the original scale was ρϲ = .94 [57].

#### Neuropsychological tests assessing executive functions (EFs), general cognitive abilities, verbal fluency, and sustained attention

##### Stop-Signal Task

We use the Stop-Signal Task by Verbruggen, Logan, and Stevens [58] to assess response inhibition. Participants are instructed to react as fast as possible to a primary stimulus in this paradigm. However, a stop signal occurs as a secondary stimulus in 25% of the trials, indicating that the reaction should be inhibited. If the reaction is inhibited correctly, the time between the presentation of primary and secondary stimulus is delayed by 50 ms for the next trial. If the reaction is not inhibited, the presentation of the secondary stimulus decreases by 50 ms. The range of delay between presentation of primary and secondary stimulus is 150–550 ms. The Stop-Signal Task has been shown to reliably measure response inhibition in children with ADHD [59].

##### Corsi Block Tapping Task

The Corsi Block Tapping Task [60] is used in a computerized version from PEBL [61, 62] in both its forward and backward versions to assess visuo-spatial working memory capacity. Participants are asked to remember a series of locations that are presented on a computer screen. At the beginning of each trial, the participant sees nine blue blocks on the screen. Then one block after another lights up in yellow for 1000 ms until the sequence length is reached. Starting with a sequence length of 2, the task consists of two trials with the same sequence length presented to the participant. If at least one sequence of the two is replicated correctly by clicking on the blocks on the screen with a mouse, the sequence length increases by 1 for the next block. In the backward task, the subject must click the blocks in the reverse order of presentation. If both tasks are not replicated correctly, the test ends. Interstimulus intervals (ISIs) and intertrial intervals are set to 1000 ms. Data on the psychometric quality of the test are available for a version using three items for each sequence length. The reliability of this version is high, with *r* = .95 [63].

##### Digit span task (WISC-IV)

The digit span task from the Wechsler Intelligence Scale for Children (fourth edition) (WISC-IV) [64], in both its forward and backward versions, is used to assess verbal working memory. Reliability of the digit span task is reported to be *r* = .76 for the backward and *r* = .84 for the forward version [64]. Hence, the digit span task from the WISC-IV is an appropriate instrument to measure verbal working memory in the study sample.

##### Verbal fluency task (VFT)

The verbal fluency task (VFT) used in this study was developed in the research group Psychophysiology and Optical Imaging at the Department of Psychiatry and Psychotherapy of the University of Tübingen and is based on the Regensburger Wortflüssigkeits-Test (RWT) [65]. Data from a NIRS measurement are recorded while the participant completes this task to assess differences in cortical brain activation resulting from the therapy. The VFT assesses semantic and phonetic fluency as well as semantic memory and consists of three blocks with three different tasks in every block. Every task is 30 s long and is followed by a resting phase of 30 s. In the first task (phonetic fluency), participants are instructed to name nouns beginning with a given letter. They are instructed not to name proper names and they are not allowed to name a series of compound words in which one of two words always remains the same such as in bird bone, bird bath, bird call, for instance. One of the following sets of letters is randomly assigned to each of the three measurements: E, P, G, A, F, M, and K, H, R. Furthermore, the sequence of the letters is randomized to prevent sequence effects. The difficulty of finding nouns beginning with a specific letter is balanced between the groups. For the second task (semantic memory), that is, the control task, participants are instructed to name the days of the week starting with Monday, and to name approximately one day per second. In the third task (semantic fluency), participants are instructed to name nouns belonging to a given category. To each measurement, one group of categories, either ‘animals, professions, drinks’, ‘colours, clothes, hobbies’, or ‘fruits, sports, toys’, is assigned randomly. The sequence of the categories is randomized, and the difficulty of the categories is balanced between the sets of words. On the behavioural level, reproducibility of the VFT is good, with *r* = .70 within a 3-week time interval [66]. Reproducibility of brain activity as measured with fNIRS was acceptable, with *r* = .50 at a single subject level [66]. Hence, the VFT, as it is used in this study, can be expected to be an appropriate instrument to measure semantic and phonetic fluency as well as semantic memory and corresponding task-related brain activity in the study sample.

##### n-back task

The *n*-back task used in this study was developed in the research group Psychophysiology and Optical Imaging at the Department of Psychiatry and Psychotherapy of the University of Tübingen. The task assesses working memory and consists of three different conditions: a 0-back, a 1-back, and a 2-back task. The tasks are presented to the participants in nine blocks, i.e. three blocks per condition containing 15 trials each, with a 20-s resting phase between active blocks. The stimulus duration is set to 300 ms, and the ISI to 1700 ms. In the 0-back task, participants are instructed to press the space bar as quickly as possible whenever they see a certain stimulus. In the 1-back task, they should respond with the space bar when any stimulus appears twice in a row. In the 2-back task, participants are instructed to press the space bar as quickly as possible whenever the current stimulus and the second last are the same. Target stimuli always constitute 4 out of the 15 presented stimuli in each block, and blocks are presented in a randomized order. In order to construct an age-appropriate version of the *n*-back task, stimuli are in image form, i.e. a moon, ball, or house. Before the actual test begins, participants practice every condition. The conditions used for testing contain different symbols than the ones used in the actual test. Data from a NIRS and an EEG measurement are recorded while the participant accomplishes this task to assess differences in cortical brain activation resulting from the therapy.

##### Go/NoGo task

The Go/NoGo task used in this study was adapted to pictorial form from a version developed by the research group of Psychophysiology and Optical Imaging at the Department of Psychiatry and Psychotherapy of the University of Tübingen. The task assesses response inhibition and consists of eight blocks with 16 trials each. The ISI is fixed to 1150 ms, and all stimuli are presented for 350 ms. Four of the eight blocks consist of go-trials only; hence, participants are instructed to press the space bar as quickly as possible whenever they see a stimulus, i.e. randomly one of three different pictures, on the computer screen. The other four blocks are designed with 12 go- as well as 4 no-go-trials. Participants are instructed to press the space bar as quickly as possible whenever they see a go-stimulus, but to inhibit the reaction when a no-go-stimulus, i.e. a fork, appears on the screen. Blocks with only go-trials and blocks consisting of mixed trials follow each other in an alternating order, separated by a resting block of 30 s. Data from a NIRS and an EEG measurement are recorded while the participant accomplishes this task to assess differences in cortical brain activation resulting from the therapy.

##### Matrix span task (WISC-IV)

The matrix span task, taken from the WISC-IV [64], assesses general cognitive abilities. The reliability of the matrix span task is reported to be *r* = .89 [64]; hence, it is an appropriate instrument to measure general cognitive abilities in the study sample.

##### Sustained attention

The Conner’s Continuous Performance Test (CPT) from PEBL [61, 67] is used to assess sustained attention and response inhibition. This test presents 360 letters with a size of one inch to the participant one at a time on a computer screen. The letters are presented in 18 blocks with 20 letters each, and the blocks follow each other consecutively. The duration of the presentation of a letter is approximately 250 ms, while the ISI varies between 1.0, 2.0, and 4.0 s. Within every triplet of blocks, the length of the ISIs is randomly distributed. Participants are instructed to always press the space bar as quickly as possible as soon as a letter appears. However, when the letter X appears, the space bar must not be pressed. The relative occurrence of an X, which remains constant across all blocks and triplets, is fixed at 10%; hence, in 90% of all letters presented, it is any letter but an X. The test-retest reliability of the Conner’s CPT is good, with values ranging between *r* = .55 and *r* = .84 [67]. Consequently, the Conner’s CPT is an appropriate test to measure sustained attention as well as response inhibition in the study sample.

#### Academic performance

##### Mathematics

The Lernverlaufsdiagnostik Mathematik für zweite bis vierte Klassen (LVD-M 2–4) assesses math performance in German primary schoolchildren from Grades 2–4 [68]. Every participant receives a math test consisting of 24 tasks randomly selected at every measurement. Reliability has been estimated and ranges between *r* = .79 and *r* = .92 [68]. In correlation analyses with other German math tests such as the DEMAT [69–71], validity has been demonstrated. Hence, this test can reliably assess math performance in the study sample.

##### Reading and writing

The Lese- und Rechtschreibtest (SLRT-II), an advanced version of the Salzburger Lese- und Rechtschreibtest (SLRT), is used to assess reading and writing skills in schoolchildren from Grades 1 to 5 (1–6 for the subtest for reading). Two parallel versions are available. The reliability coefficients for the parallel tests for reading skills range between *r* = .90 and *r* = .98. For the tests of writing skills, the interrater reliability is very high, with *r* = .998. The test-retest reliability for the writing test is between *r* = .80 and *r* = .97 with the second measurement taken 5 weeks after the first. Parallel test reliabilities range between *r* = .69 and *r* = .85 for Grades 1–4. Hence, both tests show good quality criteria and can be applied in this study.

#### Neurophysiological and other physiological measures

##### Electroencephalogram (EEG)

EEG data are collected using 22 EEG channels positioned according to the international 10–20 system. Two channels of the actiCap system (Brain Products GmbH, Germany) are used to detect horizontal eye movements and are attached 1.5 cm lateral to the outer canthus of both eyes. One additional electrode is used to detect vertical eye movement and is attached 1.5 cm below the middle of the right lower eyelid.

##### Near-infrared spectroscopy (NIRS)

NIRS is an optical imaging technique examining the blood oxygenation level-dependent (BOLD) response of brain tissue. Light from the near-infrared spectrum (700–1000 nm wavelength) can penetrate the skull and is mainly absorbed by the two chromophores oxygenated haemoglobin (oxy-Hb) and deoxygenated haemoglobin (deoxy-Hb). As the two chromophores differ in their absorption maxima, variations of the concentration of both types in the brain tissue can be derived [72]. Due to neurovascular coupling, changes in concentration of oxy- and deoxy-Hb occur in response to cortical activation [72–74]. Hence, oxy- and deoxy-Hb provide information about brain activity in respective areas [72–74]. In the present study, data are acquired with the ETG-4000 Optical Topography System (Hitachi Medical Co., Japan), which is a continuous wave system working with two different wavelengths (695 ± 20 and 830 ± 20 nm) and a temporal resolution of 10 Hz, using a 44-channel array. Relative changes of absorbed near-infrared light are transformed into concentration changes of oxy-Hb and deoxy-Hb by means of a modified Beer-Lambert law.

**Fig. 3:**
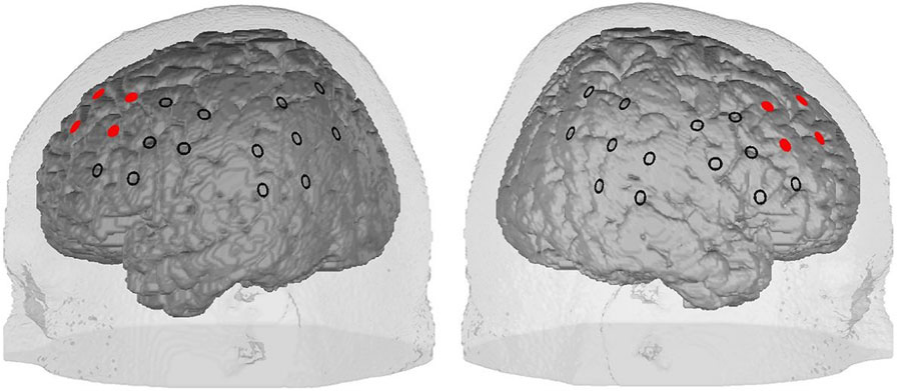
Alignment of the NIRS channels on the cortex surface. The eight channels from which the feedback signal is computed are marked in red

The 28 NIRS optodes (14 light sources (emitters), 14 detectors) are arranged in a combined NIRS/EEG cap designed to function with the Oculus Rift HMD Development Kit 2. The caps are individually localized by the EEG channels FCz and Cz according to the 10–20 system [75]. In order to assign NIRS channels that are situated in between adjacent pairs of emitters and detectors to their corresponding cortical regions, a spatial registration method of NIRS channels is applied [76]. In order to normalize the combined EEG/NIRS caps for children aged 6–10, we used the neuronavigation data of a 9-year-old girl normalized with the average brain from this age range, taken from the Template-O-Matic project [77]. A cap for a combined EEG/NIRS measurement from a previous study was placed on the girl's head. Using neuronavigation [78], optode and channel positions together with their corresponding cortical projection points on the head were obtained. The resulting coordinates from the neuronavigation were transferred to the standard Montreal Neurological Institute (MNI) space. Mapped on a virtual brain template, caps for combined EEG/NIRS measurements were customized with maximum coverage of the bilateral dlPFC (Brodmann areas 9, 46) that are used as feedback channels in the NIRS-based NFT of this study. See Fig. 3 for the alignment of the NIRS channels on the cortex surface. Hence, seven emitter and seven detector optodes are spread over prefrontal, central, temporal, and parietal areas of each hemisphere. The emitter-detector distance is 3 cm; we also employ one temporal channel on each hemisphere with a short-optode distance of 1 cm that can be used in later analysis for artefact removal (muscle artefacts as related to biting, for instance, as well as skin perfusion artefacts or other extra-cerebral signal components).

##### Electromyogram (EMG)

EMG data are collected using the BrainAmp EEG system by Brain Products. Two electrodes placed on the surface of the skin, bilaterally on the subjects’ supraspinatus muscles, measure the ratio of muscle tension between the right and left supraspinatus muscle. Reference electrodes placed on both mastoids complete the setup for the measurement. The value calculated by subtracting the normalized muscle tension of the left supraspinatus muscle from the normalized muscle tension of the right provides the feedback. Higher tension on the right will be equated to ‘activation’, higher tension on the left to ‘deactivation’.

##### Accelerometer

In this study, accelerometers are used to objectively measure motor hyperactivity at the non-dominant wrist, hip, and ankle at pre-, post-, and follow-up measurement as well as during every training session. The accelerometer used in this study is the wGT3X+ by the company ActiGraph. This device measures acceleration on the vertical, horizontal, and perpendicular axes with a range of −6 to +6 g (g = gravitational force). This small and very light sensor (5.6 cm × 3.3 cm × 1.5 cm; 19 g) is fixed to the waistband using a light belt or a clip. Furthermore, the ECGMove 3 (see below for a description of the device) measures acceleration of the torso.

##### Heart rate variability

Data from the electrocardiogram (ECG) are collected during every training session using the very light sensor ECGMove 3 (from Movisens) with a size of 62.3 mm × 38.6 mm × 11.5 mm. The sensor is fixed with two Ag/AgCl cup electrodes to the skin below the sternum. Heart rate variability can then be calculated from the ECG data.

#### Motivation

Before the start of every training session, the participants are asked to complete a questionnaire consisting of six items that assess motivation. The questionnaire was developed at the LEAD Graduate School & Research Network of the University of Tübingen. Motivation for the training session is operationalized in four dimensions: effort (i.e. “I will make an effort to do well in the training today”), joy (i.e. “I am looking forward to today’s training session”, “I only came to training because I had to”), value attributed to the training session (i.e. “I am convinced that this training session is important for me”), and importance of showing a good training performance (i.e. “It is important for me to show a good training performance”, “I am disappointed when I do not succeed in switching the lighting in the classroom on and off”).

#### Time points of assessments

**Fig. 4:**
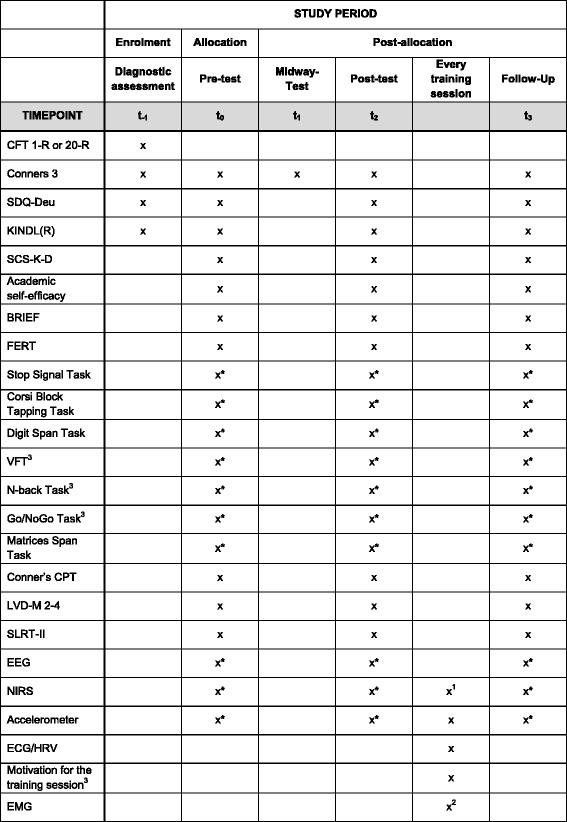
SPIRIT figure presenting an overview of the tests, questionnaires, and other methods employed at different time points in the study. ^1^If participants are assigned to one of the conditions receiving a NIRS-based NF training. ^2^If participants are assigned to the condition receiving an EMG-based BF training. ^3^These tests/questionnaires were developed in the departments of the authors of this study. *Data collection requires medication washout

The first assessment (t_−1_) takes place in order to check for all relevant inclusion and exclusion criteria. Zero to two weeks before the first training session, two baseline measurements of all relevant variables take place (t_0_). Medication washout is required for one of the two test sessions in which neuropsychological and neurophysiological measurements are applied. For an overview of the variables assessed under medication washout, see Fig. 4. After eight training sessions, ADHD symptoms are assessed in a midway test (t_1_). Zero to two weeks after the last training session, a post-test measurement of all relevant variables, again under medication washout for one of the two sessions, takes place (t_2_). Six months after the last training session, all relevant variables are again assessed in a follow-up test (t_3_).

For an overview of the tests, questionnaires, and methods employed at different time points in the study, see Fig. 4, which was designed in accordance with the standard protocol items for clinical trials [39, 40].

*BRIEF* Questionnaire assessing executive functions, *C3-P* Conners 3 parent questionnaire (long form), *C3-T* Conners 3 teacher questionnaire (long form), *CFT* Culture Fair Test, *CPT* Conner’s Continuous Performance Test, *FERT* Questionnaire to assess relevant therapy circumstances, *HRV* heart rate variability, *KINDL-R* Questionnaire to assess HRQoL, *LVD-M 2–4* Curriculum-based assessment of mathematics skills for Grades 2–4, *SCS-K-D* Brief Self-Control Scale, *SDQ-Deu* Strengths and Difficulties Questionnaire, German version, *SLRT-II* Comprehensive assessment of reading and writing skills of children, *VFT* Verbal fluency task

#### Primary outcome measures

Mean group scores of every condition will be calculated for all primary outcome measures. The C3-P and C3-T [42] assess ADHD symptoms as rated by parents and teachers at pre- (t_0_), midway (t_1_), post- (t_2_), and follow-up test (t_3_), hence allowing us to assess changes within and between conditions from pre- to midway, from pre- to post-, and from pre- to follow-up test. Furthermore, at pre- (t_0_), post- (t_2_), and follow-up test (t_3_), brain activity, i.e. the mean levels of oxy-Hb and deoxy-Hb at various channels across different cortical areas, is assessed using fNIRS data as well as EEG data from the *n*-back task assessing working memory, the Go/NoGo task assessing response inhibition, and the VFT assessing general brain activity. Consequently, changes within and between conditions in brain activity from pre- to post-, and from pre- to follow-up test can be assessed. In addition, behavioural data (i.e. mean reaction times, mean reaction time variability (*SD*), and the mean total number of commission and omission errors) are obtained from the *n*-back task and the Go/NoGo task at pre- (t_0_), post- (t_2_), and follow-up test (t_3_), hence allowing us to assess changes from pre- to post-, and from pre- to follow-up test within and between conditions.

#### Secondary outcome measures

Mean group scores of every condition will be calculated for all secondary measures. Secondary outcome measures assess diverse constructs at pre-test (t_0_), post-test (t_2_), and follow-up test (t_3_), hence allowing us to compare changes within and between conditions from pre- to post-test and from pre- to follow-up test. Children’s HRQoL is assessed using the KINDL-R questionnaires for parents and children. Parents’ satisfaction with as well as their expectations about the intervention’s effects are assessed using the FERT questionnaire [57]. Children’s mathematics, reading, and writing skills are assessed using the LVD-M 2–4 [68] and the SLRT-II [79]. Children’s self-control and academic self-efficacy are assessed using the SCS-K-D and a scale by Schwarzer and colleagues [54] in both a version for parents and one for their children. Executive functioning is furthermore assessed using a digit span task [64] (verbal working memory), the Corsi Block Tapping Task [62] (visuo-spatial working memory), and the BRIEF [56], a questionnaire handed out to parents and teachers. Sustained attention is assessed using the Conner’s Continuous Performance Test [61, 67], and response inhibition is assessed using the Stop-Signal Task [80]. General cognitive ability is assessed using the matrix span task [64]. Moreover, activity data are collected with actigraphs measuring acceleration on the vertical, horizontal, and perpendicular axes with a range of −6 to +6 g (g = gravitational force). Heart rate variability, as calculated from the ECG, as well as the motivation for every training session, as assessed with a self-report questionnaire for the children, serve as secondary outcome measures.

#### Statistics

##### Calculation of the sample size

The sample sizes for the two analytical approaches were calculated using G Power version 3.1.9.2. Firstly, we calculated the sample size that is required in order to yield a significant effect of treatment within conditions. We expect appropriate effect sizes to range between those known for within and between designs, hence expecting an effect size of *ES* = .69 [26] with a predefined α of .05 and a power of at least .80. Using a one-tailed *t* test due to directed hypotheses, the study requires at least 15 subjects per group, assuming a post- versus pre-effect, or at least 27 subjects, assuming treatment versus passive waiting control group effect. Secondly, we calculated the sample size that is required for a repeated measures analysis of variance (ANOVA) with three groups and two measurement dates in order to be able to detect effects of at least small to medium effect sizes. Hence, assuming an *ES* of .35, a predefined α of .05, a power of at least .80, and a correlation of .5 between repeated measures results in a total sample size of 84, that is, 28 per group. Consequently, taking into consideration the results of our first and second analyses, we aim for 30 participants per group.

##### Statistical evaluation of the results

For all outcome variables, we will conduct repeated measures ANOVA as well as post hoc tests. Accelerometer data will be analysed using support vector machines (Kühnhausen J, Brefeld U, Reinelt T, Gawrilow C: Using accelerometers to predict ADHD diagnoses in children, submitted) to monitor the presence of symptoms of hyperactivity. In the case that data will not be normally distributed, adequate non-parametric tests will be applied.

All data from questionnaires completed by participants, parents, and teachers who adhered to the study protocol will be included in the analyses; this also includes data from participants or informants who left the study at a certain point of time during the course of the study, i.e. after the midway test (t_1_) or after the post-test (t_2_). If data from (neuro-) psychological tests are missing, respective data from all following measurements will also be excluded from the analyses, as learning effects are expected due to participation in the respective assessments. Furthermore, data from each participant will be analysed in the participant’s respective condition (i.e. as randomized). If data from certain items of the questionnaires are missing, we will apply appropriate procedures to deal with missing values as suggested in the manual of the respective questionnaire.

##### Data security and storage

All data are acquired and stored using anonymous codes. Codes and corresponding real names are noted on a code list stored in a lockable cupboard that can only be accessed by staff members of the project. The code list will be destroyed after the data collection, including follow-up tests, is finished. All data collected will be deleted after ten years from their first publication. No data monitoring committee is required for this study, as this is not a multicentre study.

## Discussion

We presented an innovative study design and protocol of a randomized controlled trial (RCT) with NIRS-based neurofeedback training in children with ADHD. First, this study aims to investigate the specific effects of NIRS-based NFT compared to effects of EMG-based BFT on children with ADHD. Both variants of the training are conducted in a VR classroom environment. Second, we aim to compare differential effects of NIRS-based NFT in a 2D and a VR environment. Third, this study examines effects of NIRS-based NFT and EMG-based BFT on self-control as well as on school performance of children with ADHD.

There are already promising findings providing evidence for the efficacy of NIRS-based NFT in children with ADHD in the scope of a pilot study [27]. The study presented here now aims to further examine the findings in a comprehensive design. An active control condition receiving an EMG-based BFT will serve to differentiate specific as well as unspecific effects of the interventions. In addition to strong ethical concerns and poor compliance to treatment in NFTs using sham feedback as a control condition [30, 31], a control condition receiving a sham feedback is not adequate to approach this question. NFT and BFT for ADHD treatment generally train self-regulation in different domains [22, 81] in the fashion of an operant conditioning paradigm. On the one hand, participants acquire self-regulation skills that allow control of a specific endogenous parameter, namely brain activation. On the other hand, they learn to self-regulate behavioural conditions such as being attentive, sitting still, and enduring boredom. Therefore, we expect NFT as well as BFT to yield the same degree of effects in the latter domains, while only the acquisition of self-regulation skills related to the specific endogenous parameter will yield unique effects on ADHD symptomatology. Hence, comparing the effects of a NIRS-based NFT in the VR setting and those of an EMG-based BFT in VR in this study will illustrate the proportion of specific effects as well as effects common to both interventions.

With the study design presented, we furthermore aim at examining whether an NFT in a naturalistic VR setting might yield greater effects than an NFT in a 2D setting. From a theoretical point of view, both the acquisition of self-regulation skills in the laboratory and their transfer to everyday life situations (e.g. a classroom setting) might be facilitated by training in a naturalistic VR environment [34]. The VR environment elicits psychological and behavioural responses that would similarly occur in real life [34]. As these responses occur within a therapeutic setting, they provide the starting point for behavioural and psychological interventions [34]. Transfer of skills acquired in the training is furthermore facilitated due to the high degree of realism of the training setting [35]. In addition, training motivation has been identified as an important moderator of the efficacy of cognitive training, as it fosters cognitive control and prefrontal activity [35]. Training motivation may be increased by a personalized context that links the goals of the training to everyday life [35]. Hence, as a naturalistic VR environment, such as a VR classroom, links goals of the training to a real-life situation, we should expect NFT and BFT taking place in a naturalistic VR environment to yield larger effects than training in 2D. The present study will investigate whether effects of a NIRS-based NFT are larger when the training is conducted in a naturalistic VR environment compared to a 2D setting.

Children with ADHD experience poor school performance [6–8] as well as a core deficit in self-control [3], that is, “the deliberate, conscious, effortful subset of self-regulation” [82]. However, aspects often neglected in prior studies include the effects of NFT and BFT on self-control and school performance. Depicting NFT and BFT as interventions that train the exertion of self-regulation in two domains, namely self-regulating an endogenous parameter and self-regulation of behaviour, it seems plausible to expect effects on and to assess self-control while investigating effects on the self-regulation of brain activity and behaviour. Furthermore, as poor school performance is related to difficulties in behaviour, EF, and self-control [8], and both NFT and BFT have been shown to improve behaviour and EF (e.g. [83, 84]), we should expect all three interventions administered in this study to improve school performance. Hence, it is vital to assess the effects of a NIRS-based NFT and an EMG-based BFT on school performance.

The present study is limited in that participants, parents, and the trainers administering the intervention are not blinded — even though an official debriefing of parents and children as well as communication of individual results will only take place after finishing the study, hence, after the follow-up test is completed. First, participants, parents, and trainers are not blinded due to time constraints that make it impossible to use both the NIRS machine and the EMG equipment simultaneously. Second, participants either wear or do not wear the HMD, and hence they will know whether it is the 2D or VR condition they belong to. Third, as measurements during training sessions require constant observation of the data being recorded, it is impossible to blind trainers for the kind of intervention administered. However, participants, parents, and trainers are informed that every participant receives a potent intervention. Hence, we hope that bias is reduced to a minimum.

We have presented the design and protocol for a randomized controlled trial on a NIRS-based NFT in a VR classroom for children with ADHD. In addition to assessing the effects of an NFT using this relatively new technology, and besides the fact that this is, to our knowledge, the first study examining differential effects of an NFT in children with ADHD in a 2D and a VR setting, we add the assessment of concepts that have rarely been considered in prior NFT studies to established measures.

### Trial status

The trial is ongoing.

## Additional file

Additional file 1: SPIRIT checklist. (PDF 480 kb)

## Abbreviations

ADHD: Attention-deficit/hyperactivity disorder
BFT: Biofeedback training
BRIEF: Behaviour Rating Inventory of Executive Function
C3-P: Conners 3 parent questionnaire (long form)
C3-T: Conners 3 teacher questionnaire (long form)
CFT: Culture Fair Test
CPT: Conner’s Continuous Performance Test
deoxy-Hb: Deoxygenated haemoglobin
dlPFC: Dorsolateral prefrontal cortex
DSM-5: Diagnostic and Statistical Manual of Mental Disorders, fifth edition
EEG: Electroencephalogram
EF: Executive function
EMG: Electromyogram
FERT: Questionnaire to assess relevant therapy circumstances
fMRI: Functional magnet resonance imaging
fNIRS: Functional near-infrared spectroscopy
HMD: Head-mounted display
HRQoL: Health-related quality of life
HRV: Heart rate variability
ISI: Interstimulus interval
KINDL-R: Questionnaire to assess HRQoL
LVD-M 2–4: Curriculum-based assessment of mathematics skills for Grades 2–4
MNI: Montreal Neurological Institute
NFT: Neurofeedback training
NIRS: Near-infrared spectroscopy
oxy-Hb: Oxygenated haemoglobin
RTC: Randomized controlled trial
SCP: Slow cortical potential
SCS-K-D: Brief Self-Control Scale
SD: Standard deviation
SDQ-Deu: Strengths and Difficulties Questionnaire, German version
SLRT-II: Lese- und Rechtschreibtest, an advanced version of the Salzburger Lese- und Rechtschreibtest (SLRT)
VFT: Verbal fluency task
VR: Virtual reality
