# Supplementary material for: NIRS-based neurofeedback training in a virtual reality classroom for children with attention-deficit/hyperactivity disorder: study protocol for a randomized controlled trial
Source: Trials. 2017 Jan 24;18:41. doi: 10.1186/s13063-016-1769-3 (PMC5259870; doi:10.1186/s13063-016-1769-3)
Supplement: Additional file 1: — SPIRIT checklist. (PDF 480 kb) [file 13063_2016_1769_MOESM1_ESM.pdf]

SPIRIT 2013 Checklist: Recommended items to address in a clinical trial protocol and related documents*

Section/item	Item No	Description	Addressed on page number
**Administrative information**			
Title	1	Descriptive title identifying the study design, population, interventions, and, if applicable, trial acronym	1
Trial registration	2a	Trial identifier and registry name. If not yet registered, name of intended registry	4
	2b	All items from the World Health Organization Trial Registration Data Set	Information can be found throughout manuscript
Protocol version	3	Date and version identifier	4
Funding	4	Sources and types of financial, material, and other support	32-33
Roles and responsibilities	5a	Names, affiliations, and roles of protocol contributors	1-2, 33
	5b	Name and contact information for the trial sponsor	33
	5c	Role of study sponsor and funders, if any, in study design; collection, management, analysis, and interpretation of data; writing of the report; and the decision to submit the report for publication, including whether they will have ultimate authority over any of these activities	the study sponsor takes no role in any of the described fields.

	5d	Composition, roles, and responsibilities of the coordinating centre, steering committee, endpoint adjudication committee, data management team, and other individuals or groups overseeing the trial, if applicable (see Item 21a for data monitoring committee)	not applicable as there is no coordinating centre, steering committee etc. as this is not a multicentre study.
**Introduction**			
Background and rationale	6a	Description of research question and justification for undertaking the trial, including summary of relevant studies (published and unpublished) examining benefits and harms for each intervention	4-9
	6b	Explanation for choice of comparators	6
Objectives	7	Specific objectives or hypotheses	9
Trial design	8	Description of trial design including type of trial (eg, parallel group, crossover, factorial, single group), allocation ratio, and framework (eg, superiority, equivalence, noninferiority, exploratory)	10-12
**Methods: Participants, interventions, and outcomes**			
Study setting	9	Description of study settings (eg, community clinic, academic hospital) and list of countries where data will be collected. Reference to where list of study sites can be obtained	9
Eligibility criteria	10	Inclusion and exclusion criteria for participants. If applicable, eligibility criteria for study centres and individuals who will perform the interventions (eg, surgeons, psychotherapists)	10
Interventions	11a	Interventions for each group with sufficient detail to allow replication, including how and when they will be administered	10-14

	11b	Criteria for discontinuing or modifying allocated interventions for a given trial participant (eg, drug dose change in response to harms, participant request, or improving/worsening disease)	none, as participants finish the study in the allocated intervention. If they decide to quit the study participation, they are free to do so (see p. 32).
	11c	Strategies to improve adherence to intervention protocols, and any procedures for monitoring adherence (eg, drug tablet return, laboratory tests)	As described on pages 10-14, we will see participants at least twice per week for the time from pre- to post-test. In addition, the intervention takes place in the laboratory with a trainer. Consequently, it is ensured that every participant adheres to the intervention protocol.
	11d	Relevant concomitant care and interventions that are permitted or prohibited during the trial	10
Outcomes	12	Primary, secondary, and other outcomes, including the specific measurement variable (eg, systolic blood pressure), analysis metric (eg, change from baseline, final value, time to event), method of aggregation (eg, median, proportion), and time point for each outcome. Explanation of the clinical relevance of chosen efficacy and harm outcomes is strongly recommended	27
Participant timeline	13	Time schedule of enrolment, interventions (including any run-ins and washouts), assessments, and visits for participants. A schematic diagram is highly recommended (see Figure)	12; 25-26
Sample size	14	Estimated number of participants needed to achieve study objectives and how it was determined, including clinical and statistical assumptions supporting any sample size calculations	27-28

Recruitment	15	Strategies for achieving adequate participant enrolment to reach target sample size	9-10

Methods: Assignment of interventions (for controlled trials)

Allocation:			
Sequence generation	16a	Method of generating the allocation sequence (eg, computer-generated random numbers), and list of any factors for stratification. To reduce predictability of a random sequence, details of any planned restriction (eg, blocking) should be provided in a separate document that is unavailable to those who enrol participants or assign interventions	10
Allocation concealment mechanism	16b	Mechanism of implementing the allocation sequence (eg, central telephone; sequentially numbered, opaque, sealed envelopes), describing any steps to conceal the sequence until interventions are assigned	10
Implementation	16c	Who will generate the allocation sequence, who will enrol participants, and who will assign participants to interventions	10
Blinding (masking)	17a	Who will be blinded after assignment to interventions (eg, trial participants, care providers, outcome assessors, data analysts), and how	not applicable; all parties not officially blinded (p.30).
	17b	If blinded, circumstances under which unblinding is permissible, and procedure for revealing a participant's allocated intervention during the trial	not applicable; all parties not officially blinded (p.30).

Methods: Data collection, management, and analysis

Data collection methods	18a	Plans for assessment and collection of outcome, baseline, and other trial data, including any related processes to promote data quality (eg, duplicate measurements, training of assessors) and a description of study instruments (eg, questionnaires, laboratory tests) along with their reliability and validity, if known. Reference to where data collection forms can be found, if not in the protocol	10-27

	18b	Plans to promote participant retention and complete follow-up, including list of any outcome data to be collected for participants who discontinue or deviate from intervention protocols	We aim at participant retention until the follow-up test as individual results regarding effects of the intervention are only communicated after the follow-up test (p. 30). If participants do not complete the follow-up test, we still try to collect data from teacher- and parent-ratings.
Data management	19	Plans for data entry, coding, security, and storage, including any related processes to promote data quality (eg, double data entry; range checks for data values). Reference to where details of data management procedures can be found, if not in the protocol	28
Statistical methods	20a	Statistical methods for analysing primary and secondary outcomes. Reference to where other details of the statistical analysis plan can be found, if not in the protocol	28
	20b	Methods for any additional analyses (eg, subgroup and adjusted analyses)	28
	20c	Definition of analysis population relating to protocol non-adherence (eg, as randomised analysis), and any statistical methods to handle missing data (eg, multiple imputation)	As all participants receive a potent intervention, we expect no increased drop-out in any of the conditions. For missing data see p. 28.

Methods: Monitoring

Data monitoring	21a	Composition of data monitoring committee (DMC); summary of its role and reporting structure; statement of whether it is independent from the sponsor and competing interests; and reference to where further details about its charter can be found, if not in the protocol. Alternatively, an explanation of why a DMC is not needed	no data monitoring committee necessary as this is not a multicentre study.
	21b	Description of any interim analyses and stopping guidelines, including who will have access to these interim results and make the final decision to terminate the trial	not applicable as we expect no harm from the interventions. Consequently, there are no stopping guidelines.
Harms	22	Plans for collecting, assessing, reporting, and managing solicited and spontaneously reported adverse events and other unintended effects of trial interventions or trial conduct	14
Auditing	23	Frequency and procedures for auditing trial conduct, if any, and whether the process will be independent from investigators and the sponsor	No auditing committee is available as this is no multicentre study.
**Ethics and dissemination**			
Research ethics approval	24	Plans for seeking research ethics committee/institutional review board (REC/IRB) approval	32
Protocol amendments	25	Plans for communicating important protocol modifications (eg, changes to eligibility criteria, outcomes, analyses) to relevant parties (eg, investigators, REC/IRBs, trial participants, trial registries, journals, regulators)	not applicable; there will be no modifications to the running trial.
Consent or assent	26a	Who will obtain informed consent or assent from potential trial participants or authorised surrogates, and how (see Item 32)	32
	26b	Additional consent provisions for collection and use of participant data and biological specimens in ancillary studies, if applicable	not applicable as there are no ancillary studies.
Confidentiality	27	How personal information about potential and enrolled participants will be collected, shared, and maintained in order to protect confidentiality before, during, and after the trial	28

Declaration of interests	28	Financial and other competing interests for principal investigators for the overall trial and each study site	32
Access to data	29	Statement of who will have access to the final trial dataset, and disclosure of contractual agreements that limit such access for investigators	as this is no multicentre study, everyone will have full access to the final trial dataset.
Ancillary and post-trial care	30	Provisions, if any, for ancillary and post-trial care, and for compensation to those who suffer harm from trial participation	not applicable as no harm to participants is expected.
Dissemination policy	31a	Plans for investigators and sponsor to communicate trial results to participants, healthcare professionals, the public, and other relevant groups (eg, via publication, reporting in results databases, or other data sharing arrangements), including any publication restrictions	not applicable as this is no multicentre study.
	31b	Authorship eligibility guidelines and any intended use of professional writers	not applicable as this is no multicentre study.
	31c	Plans, if any, for granting public access to the full protocol, participant-level dataset, and statistical code	32
**Appendices**			
Informed consent materials	32	Model consent form and other related documentation given to participants and authorised surrogates	attached
Biological specimens	33	Plans for collection, laboratory evaluation, and storage of biological specimens for genetic or molecular analysis in the current trial and for future use in ancillary studies, if applicable	not applicable as no biological specimens are collected.

*It is strongly recommended that this checklist be read in conjunction with the SPIRIT 2013 Explanation & Elaboration for important clarification on the items. Amendments to the protocol should be tracked and dated. The SPIRIT checklist is copyrighted by the SPIRIT Group under the Creative Commons "Attribution-NonCommercial-NoDerivs 3.0 Unported" license.

Klinik für Psychiatrie und Psychotherapie mit Poliklinik
Abt.: Allgemeine Psychiatrie und Psychotherapie mit Poliklinik
Ärztlicher Direktor Prof. Dr. A. J. Fallgatter

Ansprechpartnerin:
Dr. Ann-Christine Ehlis, Dipl.-Psych.
Telefon: 07071/ 29-87103
E-Mail: ann-christine.ehlis@med.uni-tuebingen.de

Projektleiterinnen: Dr. Ann-Christine Ehlis, Dipl.-Psych. *(Klinik für Psychiatrie und Psychotherapie mit Poliklinik, AG Psychophysiologie & Optische Bildgebung)*

Prof. Dr. Caterina Gawrilow, Dipl.-Psych. *(Institut für Psychologie der Eberhard Karls Universität Tübingen, AB Schulpsychologie)*

Biofeedbacktraining bei Schulkindern mit einer Aufmerksamkeitsdefizit-/ Hyperaktivitätsstörung (ADHS): Differenzielle Effekte eines Trainings in einer 2D- oder 3D-Umgebung

- Elterninformation -

Liebe Eltern,

vielen Dank für Ihr Interesse an der Studienteilnahme. Wir gehen davon aus, dass Ihr Kind an ADHS leidet, eine abschließende Diagnostik steht aber noch aus. Diese wird aber nur dann durchgeführt, sofern Sie an der im Folgenden beschriebenen Studie teilnehmen möchten und hierzu Ihre schriftliche Einwilligung erklären. Für diese Studie suchen wir 90 Kinder mit einer ADHS.

Ihre persönlichen Daten/ Kontaktdaten werden wir nur dann weiterhin speichern, wenn sich die Diagnose ADHS bei Ihrem Kind bestätigt und wir Ihr Kind in die Studie einschließen können und alle Inklusionskriterien erfüllt sind. Sollten wir Ihr Kind nicht in die Studie einschließen können oder sollte Ihr Kind die Studienteilnahme vorzeitig beenden oder vorzeitig aus der Studie ausscheiden, so werden wir diese Daten umgehend löschen.

Nachfolgend wollen wir Ihnen die Ziele und den Ablauf der Studie erläutern.

1. Warum wird diese Studie durchgeführt?

Ziel eines jeden Biofeedbacktrainings ist, dass der Proband/ die Probandin die Kontrolle über ein bestimmtes körpereigenes Signal erhält und lernt, dieses mittels individueller Strategien zu beeinflussen. Dazu wird in jeder Trainingssitzung das körpereigene Signal gemessen und aufgezeichnet. Dieses Signal wird dann über das Sehen oder das Hören rückgemeldet und ein Proband erkennt dann Strategien, wie dieses körpereigene Signal beeinflusst werden kann. In dieser Studie verwenden zum einen ein Signal der Muskelspannung der Schulter- und Nackenmuskulatur und zum anderen ein Signal des Gehirns, welches uns Informationen über seine Durchblutung liefert.

Ziel dieser Studie ist es, zu untersuchen, wie sich diese zwei verschiedene Arten eines Biofeedbacktrainings auf die ADHS-Symptomatik und auf die schulische Leistungsfähigkeit von Kindern mit ADHS auswirken. Verschiedene Studien konnten in der Vergangenheit belegen, dass beide in dieser Studie verwendeten Arten eines Biofeedbacktrainings zu einer Verbesserung der Symptome der ADHS führen. Die Effekte solcher Trainings auf die schulische Leistungsfähigkeit wurden bisher nicht untersucht.

Nach unserem Wissen wurden solche Biofeedbacktrainings bisher nur in 2D Trainingssituationen durchgeführt. Das bedeutet, dass Probanden Aufgaben lösen, die Ihnen auf einem Computerbildschirm angezeigt werden. Wir wollen nun untersuchen, ob es einen Unterschied macht, ob man in einer 2D oder 3D Trainingssituation trainiert. Eine 3D Trainingssituation wird bei uns durch eine spezielle Brille erzeugt, die Ihr Kind aufsetzt. Mit dieser Brille fühlt es sich, wie wenn es in einer virtuellen (durch einen Computer erzeugten) Realität ist. Diese virtuelle Realität stellt in unserem Training ein Klassenzimmer dar. Die 2D Trainingssituation ist ebenso ein Klassenzimmer, Ihr Kind sitzt hierbei vor einem normalen Computerbildschirm.

Ihr Kind wird also vor Beginn des Biofeedbacktrainings einer der beiden Biofeedbacktrainings und entweder einer 2D oder 3D Trainingssituation zugelost.

2. Wie ist der Ablauf der Studie und was muss ich bei der Teilnahme beachten?

Nach dem heutigen Aufklärungsgespräch werden Sie ausreichend Zeit haben, gemeinsam mit Ihrem Kind die Studienteilnahme nochmals zu überdenken. Wenn Sie sich für eine Teilnahme entscheiden, so wird es notwendig sein, eine Diagnostiksitzung zu absolvieren.
Für die Diagnostiksitzung haben Sie zwei Optionen, die im Folgenden erläutert werden, und zwischen denen Sie sich völlig frei entscheiden können.

<u>Option 1):</u> Sie absolvieren die Diagnostiksitzung **einmalig** im Rahmen der **Biofeedback-Studie**. Dabei werden Sie jedoch keinen Arztbrief erhalten, den Sie beispielsweise dem Kinderarzt oder dem behandelnden Kinder- und Jugendpsychiater weiterleiten können. Der Zeitaufwand beträgt **einmalig ca. 1 Stunde**.

<u>Option 2):</u> Sie absolvieren die Diagnostiksitzung **einmalig** in der **Spezialambulanz ADHS** der **Kinder-und Jugendpsychiatrie** Tübingen. Sie erhalten einen Arztbrief und Beratung für die Therapie der ADHS und möglicher weiterer psychiatrischer Erkrankungen. Sollten wir es für sinnvoll erachten, dass eine Diagnostik in der Kinder- und Jugendpsychiatrie stattfindet, so werden wir Ihnen Option 2) empfehlen.

Wenn die Diagnose ADHS durch uns oder durch die Kollegen der Kinder- und Jugendpsychiatrie bestätigt ist, und alle Einschlusskriterien erfüllt sind, können wir Ihr Kind in die Studie einschließen. Sollte eines der Ausschlusskriterien erfüllt sein, so können wir Ihr Kind leider nicht in die Studie aufnehmen.

Nach Aufnahme in die Studie wird Ihr Kind einer der beiden Arten des Biofeedbacktrainings, sowie der 2D- oder 3D-Bedingung zugelost.

Direkt vor dem Beginn der Trainingssitzungen wird Ihr Kind zwei Sitzungen eines Prä-Tests absolvieren und zwei Sitzungen eines Post-Test direkt nach Beendigung der 15. Trainingssitzung. Sechs Monate nach Trainingsende folgen zwei Sitzungen eines Follow-Up Test. Beide Sitzungen dieser Tests dauern immer ca. 2,5 Stunden. Für <u>jeweils die längere der beiden Sitzungen</u> ist es, sofern Ihr Kind ein Präparat mit Methylphenidat als Wirkstoff einnimmt (z.B., Concerta®, Equasym®, Medikinet®, Ritalin® etc.), notwendig, dass Ihr Kind das Medikament für mind. 48 Stunden absetzt. Nimmt ihr Kind ein Präparat mit Atomoxetin als Wirkstoff (z.B., Strattera), so ist kein Absetzen erforderlich.

Prä-, Post- und Follow-Up-Test.

Beim Prä-, Post- und Follow-Up Test werden wir die Leistung Ihres Kindes bei verschiedenen Aufgaben untersuchen. Während drei dieser Aufgaben werden wir eine Nahinfrarotspektroskopie (NIRS)- und eine Elektroenzephalographie (EEG)-Untersuchung durchführen (diese Untersuchungen finden nur an einer der beiden Testsitzungen statt). Bei der Nahinfrarotspektroskopie wird die Aktivität verschiedener Bereiche des Gehirns mithilfe von rotem Licht gemessen: kleine Messknöpfchen, die sogenannten Optoden, senden rotes Licht aus, welches durch die Schädeldecke ins Gehirn gelangt und dort reflektiert wird. Empfänger-Optoden nehmen das reflektierte Licht auf. Über das reflektierte Licht lässt sich dann die Aktivität des Gehirns berechnen. Bei der EEG-Untersuchung werden Signale aufgezeichnet, die durch die Aktivität von Nervenzellen im Gehirn entstehen. Kleine, ringförmige Knöpfchen, die direkt auf die Kopfhaut gelegt werden, messen diese Aktivität. Weitere Informationen zur verwendeten Technik folgen auch im nächsten Kapitel. Außerdem wird Ihr Kind einen Lese- und Rechtschreibtest sowie einen Mathematiktest absolvieren.

Für den Prä-, Post- und Follow-Up Test und sowie einen Midway-Test nach der 8. Trainingssitzung (Ihr Kind wird beim Midway-Test nicht untersucht) ist auch Ihre Mithilfe gefragt. Sie werden zu den jeweiligen Zeitpunkten Fragebögen erhalten, die Informationen über das Verhalten Ihres Kinds erfragen. Das Ausfüllen der Fragebögen dauert jeweils ca. 45 Minuten.

Der weitere Ablauf sieht ein Trainingsprogramm mit 15 Trainingssitzungen à 1 Stunde (2-3 Sitzungen pro Woche) vor. Nach acht Trainingssitzungen macht Ihr Kind eine 2-3-wöchige Trainingspause, in der keine Trainingssitzungen stattfinden. Am Ende der Trainingssitzungen 6, 7 und 8 wird ihr Kind kleine Kärtchen kennen lernen, welche das „Klassenzimmer" des Biofeedbacktrainings abbilden. Mithilfe dieser Karten soll Ihr Kind lernen, in einer Art Trockenübung (ohne den Computer und ohne Rückmeldung) seinen Körper so zu aktivieren, wie es das in den Trainingssitzungen gelernt hat. Ihr Kind soll also auch außerhalb des Biofeedbacktrainings und ohne die dafür gelernten Geräte Signale seines Körpers regulieren. Diese Regulierung anhand der Karten soll auch während der Pause weiter geübt werden und beispielsweise vor dem Erledigen der Hausaufgaben oder auch in der Schule vor Situationen, die Aufmerksamkeit erfordern, erfolgen. Diese Übungen mit den Karten sollen mindestens einmal täglich erfolgen, Ihr Kind soll ca. 5-8 Mal versuchen, die Signale seines Körpers zu aktivieren und sich so in einen aufmerksameren Zustand versetzen. Diese Übung nimmt nur wenige Minuten (ca. 3-5 Minuten) in Anspruch. Da diese „Aktivierung" Konzentration und Ruhe erfordert, empfehlen wir Ihnen, diese Übungen mit Ihrem Kind alleine durchzuführen.

Der Ablauf der Studie ist in der folgenden Grafik dargestellt.

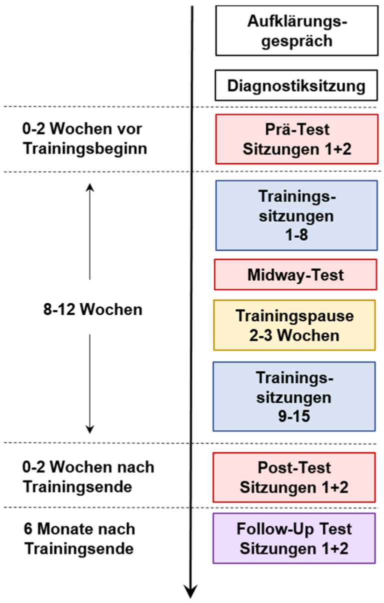

3. Der Ablauf einer Trainingssitzung

Wenn Ihr Kind zur Trainingssitzung kommt, so wird es in einem bequemen Stuhl Platz nehmen. Wurde Ihr Kind zur 3D-Bedingung zugelost, dann wird es eine Brille aufsetzen, durch welche das Kind das virtuelle Klassenzimmer sieht. Wurde Ihr Kind der 2D-Bedingung zugelost, dann wird Ihr Kind keine solche Brille aufsetzen, sondern vor einem Bildschirm Platz nehmen.
Wurde Ihr Kind dem Gehirn-Training (NIRS-Biofeedbacktraining) zugewiesen, dann werden wir im Folgenden die NIRS-Optoden auf dem Kopf platzieren. Im Fall, dass Ihr Kind am Muskel-Training (EMG-Biofeedbacktraining) teilnimmt, werden wir kleine Elektroden, welche die Aktivität der Muskulatur aufzeichnen, auf die Haut an seinen Schultern und hinter den Ohren kleben. Diese vorbereitenden Maßnahmen nehmen ca. 10-15 Minuten in Anspruch.
Zudem werden wir am Hand- und Fußgelenk und der Hüfte Ihres Kindes einen Bewegungsmesser montieren, um Bewegungen während des Trainings aufzuzeichnen. Ein EKG werden wir messen, indem wir einen kleinen Sensor unter das Brustbein des Kindes kleben. Über das EKG lässt sich ein Maß für die Trainingsmotivation Ihres Kindes berechnen. Dann startet das Training und Ihr Kind versucht mit seinen individuellen Techniken die Helligkeit des Lichts im Klassenzimmer zu steigern oder zu reduzieren, entsprechend der Anweisungen auf dem Bildschirm (Pfeil hoch = Helligkeit steigern, Pfeil runter = Helligkeit reduzieren). Jede Trainingssitzung dauert ca. 40 Minuten.

Im Anschluss an die Sitzungen Nummer 6-15 wird Ihr Kind noch kurze Übungen mit den oben beschriebenen Karten, die das Klassenzimmer abbilden, absolvieren.

4. Die verwendete Technik

Während der Studie verwenden wir bei Ihrem Kind einige Techniken zur Messung körpereigener Signale. Diese sind im Folgenden aufgeführt und erklärt.

Die Nahinfrarotspektroskopie (NIRS)

Die Nahinfrarotspektroskopie (NIRS) ist ein Untersuchungsverfahren, mit dem die Durchblutung des Gehirns gemessen werden kann, ohne dass in das Gehirn selbst eingegriffen werden muss. Dazu werden kleine „Messknöpfchen“ (Optoden), die in einer Gummiplatte integriert sind, an die Kopfhaut gelegt und mit einem Band befestigt. Mit diesem Messinstrument wird nun unschädliches Licht genau festgelegter Wellenlänge ausgesendet und die Menge an reflektiertem Licht gemessen. Anhand dieses Messergebnisses können dann Durchblutungsänderungen des Gehirns errechnet werden, die einen Hinweis auf die Hirnaktivität geben.

Die NIRS wird während einer der beiden Sitzungen des Prä-, Post- und Follow-Up Tests eingesetzt. Zudem wird die NIRS in jeder einzelnen Trainingssitzung eingesetzt, sollte Ihr Kind der Gruppe zugelost werden, die ein NIRS-basiertes Biofeedbacktraining erhält.

Zum Elektroenzephalogramm (EEG)

Das EEG ist ein Verfahren zur Messung der Gehirnaktivität. Bei Aktivität des Gehirns entstehen auf natürliche Art und Weise Spannungsänderungen, die auch an der Kopfhaut messbar sind. Das EEG misst diese Veränderungen, indem kleine Elektroden auf die Kopfhaut aufgelegt werden. Die Fixierung der Elektroden erfolgt über das Aufziehen einer Kappe, in welche die Elektroden eingearbeitet sind. Dann ist an den Auflagestellen der Elektroden zunächst eine vorsichtige Reinigung der Kopfhaut notwendig und die Leitfähigkeit zwischen Kopfhaut und Elektrode wird durch das Aufbringen einer Paste verbessert. Danach kann ein Computer das EEG aufzeichnen.

Ein EEG werden wir, in Kombination mit der NIRS, während des Prä-, Post- und Follow-Up Tests aufzeichnen.

Zum Elektromyogramm (EMG)

Analog zu Spannungsänderungen die durch Gehirnaktivität entstehen, entstehen auch bei der Aktivität von Muskeln Spannungsänderungen, die man an der Hautoberfläche über den jeweiligen Muskeln messen kann. Nach der vorsichtigen Reinigung der Haut werden Elektroden aufgeklebt und ein Computer kann das EMG aufzeichnen.

Sofern Ihr Kind der Gruppe zugelost wird, die das EMG-Biofeedbacktraining erhält, wird während jeder der 15 Trainingssitzungen ein EMG aufgezeichnet. Im Fall, dass Ihr Kind am NIRS-basierten Biofeedbacktraining teilnimmt, wird zu keinem Zeitpunkt ein EMG aufgezeichnet.

Zum Elektrokardiogramm (EKG)

Das EKG dient der Aufzeichnung der Spannungsänderungen, die sich im Körper während des Herzschlags ergeben. Sie sind für uns eine Information über die Motivation, mit der Ihr Kind das Training absolviert. Für die Aufzeichnung des EKG werden wir zwei kleine Elektroden unter dem Brustbein auf die Haut am Bauch kleben. Ein Sensor kann dann das EKG aufzeichnen.

Bei Ihrem Kind wird, unabhängig davon ob es ein EMG- oder NIRS-basiertes Biofeedbacktraining erhält, in jeder der 15 Trainingssitzungen ein EKG aufgezeichnet.

Zum Bewegungsmesser (Accelerometer)

Der Bewegungsmesser ist ein sehr kleines Gerät (5.6 cm x 3.3 cm x 1.5 cm; 19 g), welches Bewegungen aufzeichnet. Während der Sitzungen werden wir das Gerät mit einem leichten Band an Hand-, und Fußgelenk Ihres Kindes sowie an der Hüfte befestigen.

Ihr Kind wird den Bewegungsmesser an Hüfte, Hand- und Fußgelenk in jeder der 15 Trainingssitzungen tragen und zu jeweils der ersten Sitzung des Prä-, Post- und Follow-Up-Tests.

Das NIRS-, das EEG-, das EKG- und das EMG-Gerät sowie der Bewegungsmesser halten alle für die Sicherheit des Betriebes und insbesondere die Sicherheit von Probanden und Patienten erforderlichen Grenzwerte ein. Beide Geräte werden in regelmäßigen Abständen gewartet und auf ihre Sicherheit geprüft. Ionisierende Strahlen wie Röntgenstrahlen, radioaktive Strahlen oder schädliches Licht werden nicht verwendet.

Risiken/ Nebenwirkungen der Biofeedbacktrainings

Das NIRS-basierte Biofeedbacktraining kann in seltenen Fällen einen durch Muskelspannung induzierten, migräneartigen Kopfschmerz auslösen. Selten wird auch von Hitzewallungen oder leichter Angst berichtet. Bei einem EMG-basierten Biofeedbacktraining kann es vorrübergehend zu leichten Verspannungen oder leichtem Muskelkater kommen.

Während der Exposition in der virtuellen Realität trägt Ihr Kind einen Bildschirm vor den Augen, der das gesamte Gesichtsfeld abdeckt. Es sieht ein Klassenzimmer mit Klassenkameraden und einem Lehrer vor einer Tafel. Abgesehen von vorübergehenden Nebenwirkungen wie Übelkeit, Schwindel und verschwommenem Sehen sind durch die Exposition in der virtuellen Realität keine langfristigen Nebenwirkungen zu erwarten.

5. Zur Einbeziehung des Klassenlehrers/ der Klassenlehrerin in die Studie

Bei der Diagnose der ADHS und Ihrer Beurteilung spielt der Klassenlehrer bzw. die Klassenlehrerin Ihres Kindes eine wichtige Rolle. Daher wäre es für uns sehr wichtig, dass der Klassenlehrer/ die Klassenlehrerin die ADHS-Symptome und die Leistungsfähigkeit Ihres Kindes für uns beurteilt, insofern er/ sie damit einverstanden ist. Hierzu würden wir Ihnen zum Zeitpunkt der Diagnostiksitzung zunächst eine Einverständniserklärung und die ersten Fragebögen für den Klassenlehrer/ die Klassenlehrerin Ihres Kindes mitgeben, die Sie ihm/ihr bitte aushändigen. Wir würden es sehr begrüßen, wenn Sie das persönliche Gespräch mit dem Klassenlehrer/ der Klassenlehrerin suchen und ihm/ihr über die Studienteilnahme Ihres Kindes berichten und ihm/ihr in diesem Zuge die Unterlagen zukommen lassen. Der Klassenlehrer/ die Klassenlehrerin würde dann, wenn er/sie einverstanden ist zu drei weiteren Zeitpunkten (Midway-, Post- und Follow-Up Test) Fragebögen von uns erhalten.

Der Klassenlehrer/die Klassenlehrerin wird, wenn er/sie sich bereit erklärt einen Beitrag zur Studie zu leisten, zwischen Fragebögen in Papierform und Online-Fragebögen wählen können. Sofern er/sie die Papierform wünscht, werden wir Ihnen bzw. Ihrem Kind jeweils einen Briefumschlag mit den Fragebögen und einem frankierten Rückumschlag mitgeben, den Sie bzw. Ihr Kind dann bitte umgehend dem Klassenlehrer/ der Klassenlehrerin mit der Bitte um Ausfüllen aushändigen.

Wir werden den Klassenlehrer/ die Klassenlehrerin nur mit Ihrem Einverständnis kontaktieren. Indem Sie Ihr Einverständnis zur Studienteilnahme geben, sehen wir dieses Einverständnis als ebenso gegeben.

6. Ihr Nutzen von der Teilnahme an dieser Studie

Wir gehen davon aus, dass es durch beide untersuchten Methoden des Biofeedbacktrainings zu einer deutlichen Reduktion der ADHS-Symptomatik Ihres Kindes kommt.

7. Entstehen für mich Kosten durch die Teilnahme an der Studie? Erhalte ich eine Aufwandsentschädigung?

Ihnen entstehen durch die Teilnahme an der Studie keinerlei Kosten, außer Ihre Kosten für An- und Abreise und ggf. Parkgebühren.

8. Kann meine Teilnahme an der Studie vorzeitig beendet werden?

Sie können jederzeit, auch ohne Angabe von Gründen, Ihre Teilnahme beenden, ohne dass Ihnen dadurch irgendwelche Nachteile entstehen.

9. Was geschieht mit meinen Daten bzw. den Daten meines Kindes?

Wir gewährleisten die vertrauliche Behandlung aller Daten. Damit ist auch gewährleistet, dass bei der wissenschaftlichen Veröffentlichung der Ergebnisse keine Zuordnung der Befunde zur Ihrer Person oder zur Person Ihres Kindes möglich ist.
Die Speicherung sämtlicher Daten erfolgt in verschlüsselter Form unter Verwendung anonymer Codes. Alle im Verlauf der Studie erhobenen Daten werden nach der Auswertung 10 Jahre aufbewahrt. Alle Unterlagen, über die eine personenbezogene Zuordnung der erhobenen Daten (Probandeneinwilligungserklärung und Probandenidentifizierungsliste) möglich ist, verbleiben im Studienzentrum und sind nur den entsprechenden Studienmitarbeitern, die den Bestimmungen der Schweigepflicht unterliegen, zugänglich.

Einwilligungserklärung für die Teilnahme an der Studie

....

Name der **Eltern/ Erziehungsberechtigten** des Kindes in Druckbuchstaben

geb. am ....

Ich bin in einem persönlichen Gespräch durch

....

Name des **Aufklärenden**

ausführlich und verständlich über Wesen, Bedeutung, Risiken und Tragweite der Studie **Biofeedbacktraining bei Schulkindern mit einer Aufmerksamkeitsdefizit-/Hyperaktivitätsstörung (ADHS): Differenzielle Effekte eines Trainings in einer 2D- oder 3D-Umgebung** aufgeklärt worden. Ich habe darüber hinaus den Text der Elterninformation sowie die hier nachfolgend abgedruckte Datenschutzerklärung gelesen und verstanden. Ich hatte die Gelegenheit, mit dem Prüfer über die Durchführung der Studie zu sprechen. Alle meine Fragen wurden zufrieden stellend beantwortet.

Möglichkeit zur Dokumentation zusätzlicher Fragen seitens des Probanden oder sonstiger Aspekte des Aufklärungsgesprächs:

Ich hatte ausreichend Zeit, mich zu entscheiden.

Mir ist bekannt, dass ich jederzeit und ohne Angabe von Gründen meine Einwilligung zur Teilnahme an der Studie zurückziehen kann (mündlich oder schriftlich), ohne dass mir daraus Nachteile für meine medizinische Behandlung entstehen.

Datenschutz

Information zum Datenschutz

Ihre im Rahmen der wissenschaftlichen Untersuchung erhobenen Daten werden vertraulich behandelt und ausschließlich in verschlüsselter Form weitergegeben. Die für die wissenschaftliche Untersuchung wichtigen Daten werden in verschlüsselter (anonymisiert, ohne Namensnennung) Form in einen gesonderten Dokumentationsbogen eingetragen.
Die Zuordnung der verschlüsselten Daten zu Ihrer Person ist nur anhand einer Teilnehmerliste möglich, die in einem verschlossenen Schrank, getrennt von den Studienunterlagen aufbewahrt wird und nur den Studienleitern zugänglich ist. Alle im Verlauf der Studie erhobenen Daten werden nach der Auswertung 10 Jahre aufbewahrt.
Sollten Sie von der Studie zurücktreten, können Sie entscheiden, ob die bereits vorliegenden Daten vernichtet werden müssen oder weiterverwendet werden dürfen.

Einwilligungserklärung

Ich erkläre mich der Verwendung der im Rahmen der Studie *Biofeedbacktraining bei Schulkindern mit einer Aufmerksamkeitsdefizit-/Hyperaktivitätsstörung (ADHS): Differenzielle Effekte eines Trainings in einer 2D- oder 3D-Umgebung* erhobenen Daten in der oben beschriebenen Weise einverstanden. Ich kann jeder Zeit meine Daten beim Studienleiter einsehen.

....

Name der **Eltern/ Erziehungsberechtigten** des Kindes in Druckbuchstaben

....

Datum Unterschrift **der Eltern/ Erziehungsberechtigten**

Ich habe das Aufklärungsgespräch geführt und die Einwilligung der Eltern/ Erziehungsberechtigten eingeholt.

....

Name des **Prüfers** in Druckbuchstaben

....

Datum Unterschrift des aufklärenden **Prüfers**

Klinik für Psychiatrie und Psychotherapie mit Poliklinik
Abt.: Allgemeine Psychiatrie und Psychotherapie mit Poliklinik
Ärztlicher Direktor Prof. Dr. A. J. Fallgatter

Ansprechpartnerin:
Dr. Ann-Christine Ehlis, Dipl.-Psych.
Telefon: 07071/ 29-87103
E-Mail: ann-christine.ehlis@med.uni-tuebingen.de

Projektleiterinnen: Dr. Ann-Christine Ehlis, Dipl.-Psych. *(Klinik für Psychiatrie und Psychotherapie mit Poliklinik, AG Psychophysiologie & Optische Bildgebung)*

Prof. Dr. Caterina Gawrilow, Dipl.-Psych. *(Institut für Psychologie der Eberhard Karls Universität Tübingen, AB Schulpsychologie)*

Biofeedbacktraining bei Schulkindern mit einer Aufmerksamkeitsdefizit-/Hyperaktivitätsstörung (ADHS): Differenzielle Effekte eines Trainings in einer 2D- oder 3D-Umgebung

- Probandeninformation -

Hallo,

wir freuen uns sehr über dein Interesse an der Teilnahme an unserer Studie. Vermutlich merkst du, dass du dich in manchen Situationen schlechter als andere Kinder konzentrieren kannst, vielleicht auch unüberlegt handelst und oft unruhig bist? Das Training, was wir dir anbieten, kann dir helfen, diese Dinge zu verbessern.

Ganz wichtig: immer, wenn du Fragen hast, dann frage nach. Wir werden dir alles ganz genau erklären, bis du es verstanden hast.

1. Wie läuft die Studie ab?

Heute wollen wir dir alles rund um die Studie erklären. Frage immer nach, wenn du etwas nicht verstanden hast. Du hast nach dem heutigen Termin noch genügend Zeit, dir nochmals zu überlegen, ob du an der Studie teilnehmen willst.

Unser Training für dich

Bei unserem Training sollst du lernen, dich zu konzentrieren und aufmerksam zu sein. Dabei sollst du möglichst ruhig sitzen. Wir wollen in dieser Studie zwei verschiedene Arten von solch einem Training miteinander vergleichen. Bei der einen Art werden kleine Messknöpfchen an den Schultern und hinter den Ohren befestigt, bei der anderen Art werden einige Messknöpfchen auf deine Kopfhaut aufgelegt. An welcher Art du teilnimmst ist Zufall. Außerdem wollen wir, dass sich ein Teil der teilnehmenden Kinder wie in einem Klassenzimmer fühlt. Dazu werden manche Kinder diese Brille (zeigen!) aufsetzen. Ein anderer Teil der Kinder wird ein Klassenzimmer vor sich auf einem Bildschirm sehen und diese Brille nicht tragen. Das Ziel ist für alle Kinder, dass sie das Licht im Klassenzimmer heller oder dunkler werden lassen sollen.

Während dem Training werden wir auch deinen Herzschlag aufzeichnen und mit drei kleinen und sehr leichten Geräten, die wir an deiner Hüfte und deinem Handgelenk und Fußgelenk befestigen, deine Bewegungen erfassen.

Wenn du an der Studie teilnimmst, dann wirst du insgesamt 15 Mal zum Training zu uns nach Tübingen kommen, nach acht Mal hast du 2-3 Wochen frei. Neben den Trainingssitzungen wirst du insgesamt sechs Mal für verschiedene Tests zu uns kommen, bei der du verschiedene Aufgaben bearbeitest und wir schauen, wie gut du diese bearbeiten kannst. Die zwei ersten Testsitzungen werden sehr bald sein, noch bevor du mit dem Training beginnst. Ein drittes und viertes Mal kommst du für Tests in zwei oder drei Monaten, wenn alle Trainingssitzungen vorbei sind und ein fünftes und sechstes Mal ein halbes Jahr nach dem Ende der Trainingssitzungen.

Nach der Hälfte der Trainingssitzungen (8 Stück) hast du für 2-3 Wochen frei. In dieser Zeit sollst du, immer, wenn du dich konzentrieren musst (z.B. vor dem Erledigen von Hausaufgaben), in Gedanken versuchen, das Licht im Klassenzimmer anzumachen. Das werden wir dir erleichtern, indem wir dir kleine Erinnerungskärtchen mitgeben, die das Klassenzimmer zeigen, so, wie du es aus dem Training kennst.

2. Mögliche Nebenwirkungen des Trainings

Die eine Art von Training kann in seltenen Fällen starke Kopfschmerzen auslösen. Selten berichten Leute, die am Training teilnehmen auch davon, dass ihnen plötzlich sehr warm wird oder sie etwas Angst haben. Bei der anderen Art des Trainings können deine Muskeln an den Schultern für eine kurze Zeit etwas schmerzen und ziehen (wie beim Muskelkater).

Solltest du der Gruppe von Kindern zugelost werden, die sich wie in einem Klassenzimmer fühlen sollen und die spezielle Brille tragen werden, so kann dir vorrübergehend schwindlig oder schlecht werden oder es kann sein, dass du kurz verschwommen siehst. Diese Nebenwirkungen sollten aber schnell wieder verschwinden.

3. Dein Nutzen von der Teilnahme an dieser Studie

Wir gehen davon aus, dass es durch beide Arten des Trainings dazu führen, dass du dich besser konzentrieren kannst und weniger unruhig bist. Außerdem denken wir, dass du dein Verhalten nach dem Training besser kontrollieren kannst.

4. Was bekomme ich dafür, dass ich zum Training und zu den Tests komme?

Wenn du gut mitmachst und regelmäßig zum Training kommst, dann erhältst du Sticker, die du in deinem persönlichen Stickeralbum sammeln kannst.

5. Was ist, wenn ich absolut keine Lust mehr habe, weiter zum Training und den Tests zu kommen?

Du kannst jederzeit deine Teilnahme an der Studie beenden, ohne dass dir dadurch irgendwelche Nachteile entstehen.

Einwilligungserklärung für deine Teilnahme an der Studie

....

Name des **Kindes** in Druckbuchstaben

geb. am ....

Ich bin in einem persönlichen Gespräch durch

....

Name des **Aufklärenden**

ausführlich und verständlich über die Studie **Biofeedbacktraining bei Schulkindern mit einer Aufmerksamkeitsdefizit-/Hyperaktivitätsstörung (ADHS): Differenzielle Effekte eines Trainings in einer 2D- oder 3D-Umgebung** aufgeklärt worden. Die/der Aufklärende hat alle meine Fragen beantwortet und ich habe mit meinen Eltern über meine Studienteilnahme gesprochen.

Ich hatte ausreichend Zeit, mich zu entscheiden.

Ich weiß, dass ich jederzeit meine Teilnahme an der Studie beenden kann und, dass mir daraus kein Nachteil entsteht.

Möglichkeit zur Dokumentation zusätzlicher Fragen seitens des Probanden oder sonstiger Aspekte des Aufklärungsgesprächs:

Klinik für Psychiatrie und Psychotherapie mit Poliklinik
Abt.: Allgemeine Psychiatrie und Psychotherapie mit Poliklinik
Ärztlicher Direktor Prof. Dr. A. J. Fallgatter

Ansprechpartnerin:
Dr. Ann-Christine Ehlis, Dipl.-Psych.
Telefon: 07071/ 29-87103
E-Mail: ann-christine.ehlis@med.uni-tuebingen.de

Projektleiterinnen: Dr. Ann-Christine Ehlis, Dipl.-Psych. *(Klinik für Psychiatrie und Psychotherapie mit Poliklinik, AG Psychophysiologie & Optische Bildgebung)*

Prof. Dr. Caterina Gawrilow, Dipl.-Psych. *(Institut für Psychologie der Eberhard Karls Universität Tübingen, AB Schulpsychologie)*

Biofeedbacktraining bei Schulkindern mit einer Aufmerksamkeitsdefizit-/Hyperaktivitätsstörung (ADHS): Differenzielle Effekte eines Trainings in einer 2D- oder 3D-Umgebung

- Lehrerinformation -

Sehr geehrte Damen und Herren,

Ihr/e Schüler/in ______________________________ nimmt an unserer Studie **Biofeedbacktraining bei Schulkindern mit einer Aufmerksamkeitsdefizit-/Hyperaktivitätsstörung (ADHS): Differenzielle Effekte eines Trainings in einer 2D- oder 3D-Umgebung** teil, für welche wir 90 Kinder mit einer ADHS rekrutieren.

Die Erziehungsberechtigten des Schülers/ der Schülerin haben Ihr Einverständnis gegeben, dass wir Sie kontaktieren dürfen.

Ziel unserer Studie ist es, herauszufinden, inwiefern verschiedene Arten eines Biofeedbacktrainings Auswirkungen auf die ADHS-Symptomatik und die schulische Leistungsfähigkeit von Kindern mit ADHS haben.

Ein Biofeedbacktraining hat immer zum Ziel, dass die Teilnehmer Kontrolle über körpereigene Signale erhalten und somit Zustände wie z.B. die Aufmerksamkeit oder auch das Verhalten insgesamt besser steuern können.

Im Zuge der Studienteilnahme des genannten Schülers/ der Schülerin möchten wir Sie fragen, ob Sie bereit sind, abhängig vom Studienverlauf, zu vier bis fünf verschiedenen Zeitpunkten mittels Fragebögen Auskunft über seine/ ihre ADHS-Symptomatik, sein Verhalten sowie seine kognitiven Funktionen zu geben.

Ihre Teilnahme am Ausfüllen dieser Fragebögen ist freiwillig. Ihre Daten werden also nur dann verwendet, wenn Sie einverstanden sind und hierzu im Folgenden Ihre Einwilligung erklären.

Das Ausfüllen dieser Fragebögen würde jeweils ca. 45 Minuten in Anspruch nehmen. Sie können die Fragebögen entweder als Papierbögen, gemeinsam mit einem frankierten Rückumschlag, erhalten und der jeweilige Schüler/ die jeweilige Schülerin wird Ihnen die Fragebögen überreichen. Alternativ können Sie sich auch dazu entscheiden, die Fragebögen am Computer auszufüllen. Sollten Sie sich für die Online-Version entscheiden, dann erhalten Sie per Email einen personalisierten Link zu dem jeweiligen Fragebogen.

Sollten Sie sich für die Online-Variante der Fragebogenbeantwortung entscheiden, so können Sie einen Online-Fragebogen jederzeit und ohne Begründung abbrechen indem Sie einfach das Browserfenster schließen. Entscheiden Sie sich, die Fragebögen nicht auszufüllen, so entstehen Ihnen hieraus in der Folge keine negativen Konsequenzen.

Nachfolgend erhalten Sie noch einige Informationen zur Studie.

1. Warum wird diese Studie durchgeführt?

Ziel dieser Studie ist, zu untersuchen, wie sich zwei verschiedene Arten eines Biofeedbacktrainings zum einen auf die ADHS-Symptomatik und aber auch auf die schulische Leistungsfähigkeit von Kindern mit einer ADHS auswirken. Verschiedene Studien konnten in der Vergangenheit belegen, dass beide in dieser Studie verwendeten Arten eines Biofeedbacktrainings zu einer Verbesserung der Symptome der ADHS führen. Die Effekte solcher Trainings auf die schulische Leistungsfähigkeit wurden bisher nicht untersucht.
Zudem wurden solche Biofeedbacktrainings unserem Wissen nach bisher nur in 2D Trainingssituationen durchgeführt. Das bedeutet, dass Probanden Aufgaben lösen, die Ihnen auf einem Computerbildschirm angezeigt werden. Wir wollen daher untersuchen, ob es einen

Unterschied macht, ob man in einer 2D oder 3D Trainingssituation, also vor einem Computerbildschirm oder in einer virtuellen Realität, trainiert.

2. Wie ist der Ablauf der Studie und was muss ich bei der Teilnahme beachten?

Während der Teilnahme Ihres Schülers/ Ihrer Schülerin an unserer Studie, würden wir Sie gerne insgesamt vier Mal kontaktieren, um verschiedene Fragebögen für auszufüllen. Diese Fragebögen erfassen die ADHS-Symptomatik, die Verhaltensauffälligkeiten und –stärken des Kindes und seine/ihre kognitiven Funktionen. Wir werden Sie nur dann kontaktieren, wenn Sie hierzu im Folgenden ihr schriftliches Einverständnis geben.

Sie erhalten die Fragebögen entweder in ausgedruckter Form mit einem frankierten Rückumschlag oder, sofern Sie sich weiter unten für die Online-Version entscheiden, über einen personalisierten Link, den Sie von uns erhalten.

3. Ihr Nutzen von der Teilnahme an dieser Studie

Sie haben keinen persönlichen Nutzen durch Ihre Teilnahme an der Studie.

Wir gehen davon aus, dass es durch die untersuchten Methoden des Biofeedbacktrainings zu einer deutlichen Reduktion der ADHS-Symptomatik und des Verhaltens Ihres Schülers bzw. Ihrer Schülerin kommt. Zudem erwarten wir auch eine Verbesserung seiner/ihrer schulischen Leistungsfähigkeit. Durch Ihre Einwilligung zum Ausfüllen der Fragebögen helfen Sie uns sehr, eventuelle Verbesserungen zu erfassen.

4. Entstehen für mich Kosten durch die Teilnahme an der Studie? Erhalte ich eine Aufwandsentschädigung?

Ihnen entstehen durch die Teilnahme an der Studie keinerlei Kosten. Sie erhalten keine Aufwandsentschädigung.

5. Kann meine Teilnahme an der Studie vorzeitig beendet werden?

Sie können jederzeit, auch ohne Angabe von Gründen, Ihre Teilnahme beenden, ohne dass Ihnen dadurch irgendwelche Nachteile entstehen.

6. Was geschieht mit meinen Daten?

Wir gewährleisten die vertrauliche Behandlung Ihrer Daten. Damit ist auch gewährleistet, dass bei der wissenschaftlichen Veröffentlichung der Ergebnisse keine Zuordnung der Befunde zur Ihrer Person oder zur Person Ihres Schülers/ Ihrer Schülerin oder seiner Eltern/Erziehungsberechtigten möglich ist. Die von Ihnen übermittelten Daten werden ausschließlich unter Verwendung des anonymen Codes Ihres Schülers/ Ihrer Schülerin gespeichert.

Online-Version der Fragebögen. Die Ergebnisse der Online-Fragebogen werden nach Ihrem Ausfüllen heruntergeladen und aus dem Internet gelöscht. Bis zum Download werden Sie auf Servern in Deutschland gespeichert, die durch das Bundesamt für Sicherheit in der Informationstechnologie zertifiziert sind.

Online-Version und Papier-Version der Fragebögen. Die Speicherung sämtlicher Daten erfolgt in verschlüsselter Form unter Verwendung anonymer Codes. Alle im Verlauf der Studie erhobenen Daten werden nach der Auswertung 10 Jahre aufbewahrt. Alle Unterlagen, über die eine personenbezogene Zuordnung der erhobenen Daten (Probandeneinwilligungserklärung und Probandenidentifizierungsliste) möglich ist, verbleiben im Studienzentrum und sind nur den entsprechenden Studienmitarbeitern, die den Bestimmungen der Schweigepflicht unterliegen, zugänglich.

7. An wen kann ich mich wenden, wenn ich Fragen habe?

Sollten Sie Fragen haben, so können Sie sich jederzeit an die Mitarbeiter der Studie wenden, insbesondere an Friederike Blume. Sie erreichen sie unter friederike.blume@uni-tuebingen.de bzw. unter 07071/ 29 – 75633.

Einwilligungserklärung für das Ausfüllen der Fragebögen

…………………………………………………………………………………………..

Name des **Schülers/ der Schülerin** in Druckbuchstaben

....

Name der **Lehrerin/ des Lehrers** in Druckbuchstaben

……………………………………………………………

falls Online-Version gewünscht, **Email-Adresse**

Ich bin in mit diesem Informationsmaterial ausführlich und verständlich über Wesen, Bedeutung und Tragweite des Ausfüllens der Fragebögen für die Studie **Biofeedbacktraining bei Schulkindern mit einer Aufmerksamkeitsdefizit-/Hyperaktivitätsstörung (ADHS): Differenzielle Effekte eines Trainings in einer 2D- oder 3D-Umgebung** aufgeklärt worden. Ich habe den Text der Lehrerinformation sowie die hier nachfolgend abgedruckte Datenschutzerklärung gelesen und verstanden.

Ich hatte ausreichend Zeit, mich zu entscheiden.

Mir ist bekannt, dass ich jederzeit und ohne Angabe von Gründen meine Einwilligung zur Teilnahme am Online-Fragebogen zurückziehen kann (mündlich oder schriftlich), ohne dass mir daraus Nachteile entstehen.

Datenschutz

Information zum Datenschutz

Ihre im Rahmen der wissenschaftlichen Untersuchung erhobenen Daten werden vertraulich behandelt und ausschließlich in verschlüsselter Form weitergegeben. Die für die wissenschaftliche Untersuchung wichtigen Daten werden in verschlüsselter (anonymisiert, ohne Namensnennung) Form in einen gesonderten Dokumentationsbogen eingetragen.

Die Zuordnung der verschlüsselten Daten zu Ihrer Person nicht möglich, lediglich eine Zuordnung zur Person Ihres Schülers/Ihrer Schülerin. Diese Zuordnung ist nur über eine Probandenidentifikationsliste möglich, die in einem verschlossenen Schrank, getrennt von den Studienunterlagen aufbewahrt wird und nur den Studienleitern zugänglich ist. Alle im Verlauf

der Studie erhobenen Daten werden nach der Auswertung 10 Jahre aufbewahrt. Sollten Sie von der Studie zurücktreten wollen, können Sie entscheiden, ob die bereits vorliegenden Daten vernichtet werden müssen oder weiterverwendet werden dürfen.

Einwilligungserklärung

Ich erkläre mich der Verwendung der im Rahmen der Fragebögen für die Studie *Biofeedbacktraining bei Schulkindern mit einer Aufmerksamkeitsdefizit-/Hyperaktivitätsstörung (ADHS): Differenzielle Effekte eines Trainings in einer 2D- oder 3D-Umgebung* erhobenen Daten in der oben beschriebenen Weise einverstanden. Ich kann meine Daten zu jeder Zeit beim Studienleiter einsehen.

....

Name des **Lehrers/ der Lehrerin** in Druckbuchstaben

....

Datum Unterschrift **Lehrer/Lehrerin**
